# Novel anti-CD30/CD3 bispecific antibodies activate human T cells and mediate potent anti-tumor activity

**DOI:** 10.3389/fimmu.2023.1225610

**Published:** 2023-08-14

**Authors:** Mary L. Faber, Robyn A. A. Oldham, Archana Thakur, Mary Jo Rademacher, Ewa Kubicka, Theresa A. Dlugi, Steven A. Gifford, William M. McKillop, Nathan J. Schloemer, Lawrence G. Lum, Jeffrey A. Medin

**Affiliations:** ^1^ Department of Pediatrics, Medical College of Wisconsin (MCW), Milwaukee, WI, United States; ^2^ Department of Medical Biophysics, University of Toronto, Toronto, ON, Canada; ^3^ Department of Medicine, Division of Hematology/Oncology, University of Virginia Cancer Center, Charlottesville, VA, United States; ^4^ Department of Biochemistry, MCW, Milwaukee, WI, United States

**Keywords:** immunotherapy, cytotoxicity, tumor necrosis factor receptor superfamily, xenograft tumor models, transplantation, antibody heteroconjugation, membrane proteome array, surface plasmon resonance

## Abstract

CD30 is expressed on Hodgkin lymphomas (HL), many non-Hodgkin lymphomas (NHLs), and non-lymphoid malignancies in children and adults. Tumor expression, combined with restricted expression in healthy tissues, identifies CD30 as a promising immunotherapy target. An anti-CD30 antibody-drug conjugate (ADC) has been approved by the FDA for HL. While anti-CD30 ADCs and chimeric antigen receptors (CARs) have shown promise, their shortcomings and toxicities suggest that alternative treatments are needed. We developed novel anti-CD30 x anti-CD3 bispecific antibodies (biAbs) to coat activated patient T cells (ATCs) *ex vivo* prior to autologous re-infusions. Our goal is to harness the dual specificity of the biAb, the power of cellular therapy, and the safety of non-genetically modified autologous T cell infusions. We present a comprehensive characterization of the CD30 binding and tumor cell killing properties of these biAbs. Five unique murine monoclonal antibodies (mAbs) were generated against the extracellular domain of human CD30. Resultant anti-CD30 mAbs were purified and screened for binding specificity, affinity, and epitope recognition. Two lead mAb candidates with unique sequences and CD30 binding clusters that differ from the ADC in clinical use were identified. These mAbs were chemically conjugated with OKT3 (an anti-CD3 mAb). ATCs were armed and evaluated *in vitro* for binding, cytokine production, and cytotoxicity against tumor lines and then *in vivo* for tumor cell killing. Our lead mAb was subcloned to make a Master Cell Bank (MCB) and screened for binding against a library of human cell surface proteins. Only huCD30 was bound. These studies support a clinical trial in development employing *ex vivo*-loading of autologous T cells with this novel biAb.

## Introduction

CD30 is an excellent immunotherapy target due to its expression on multiple pediatric and adult malignancies, including Hodgkin lymphoma (HL), peripheral T cell lymphoma, cutaneous T cell lymphoma, acute myeloid leukemia, testicular germ cell tumors, and numerous other cancers (1–5). In fact, one meta-analysis reported that 24.5% of all solid tumors are CD30^+^ (6). Meanwhile, expression of CD30 on normal cells is restricted to a small subset of activated T and B lymphocytes (5, 7).

CD30^+^ malignancies are widespread. HL alone makes up ~10% of all lymphomas diagnosed in the US. HL is even more prevalent in adolescents, where it accounts for ~14% of all cancers diagnosed (8). With new therapies, the 5-year survival rate for HL has now reached ~87% (9). However, short- and long-term toxicities from conventional chemotherapy and radiation leave patients with significant morbidity and even mortality. Further, for patients with primary refractory or relapsed disease, outcomes are poor, with 5-year overall survival rates between 20%-31% (10, 11). The anti-CD30 antibody-drug conjugate (ADC) brentuximab vedotin (AC10 clone mAb) was approved by the FDA for some forms of HL in 2011 (12). Brentuximab has demonstrated clinical efficacy, with a 5-year survival of 41% in a phase 2 clinical trial of heavily pre-treated relapsed/refractory HL (13). However, only 9% of the patients remained in complete remission without requiring additional therapy (13). Brentuximab has also been used in up-front pediatric high-risk HL with promising results (14). The success of brentuximab validates CD30 as a therapeutic target for treating a variety of cancers; but the toxicities associated with brentuximab (15) and clinical limitations suggest that alternative treatment approaches/augmentations are needed.

Other immunotherapies targeting CD30 are being developed, including T cells with engineered expression of chimeric antigen receptors (CARs) (16–18) and anti-CD30 x anti-CD16A bispecific antibodies (biAbs) to engage NK cells and target tumor cells (19, 20). Both CARs and biAbs redirect effector immune cells to kill tumor-associated antigen (TAA)-expressing cancer cells in a non-MHC restricted manner. BiAbs combine specificity for T or NK cells with specificity for a TAA, resulting in an entity that can simultaneously bind both cell types (21, 22). BiAbs can be infused directly into the patient or used to coat (arm) effector cells *ex vivo* that are then re-infused (21, 22). BiAb therapies have been successfully applied to a range of malignancies, including breast cancer, glioma, carcinoma, and lymphoma (23–26).

We developed novel and unique anti-CD30 x anti-CD3 biAbs that trigger T cell-mediated activation and elimination of CD30-expressing tumor cells. Clones 8D10 and 10C2, which have unique amino acid sequences and bind epitopes on CD30 different from AC10 and from each other, were selected for heteroconjugation with the anti-CD3 mAb OKT3. Human activated T cells (ATCs) armed with our 8D10 biAb are effective at lysing CD30^+^ tumor cells and secrete an array of pro-inflammatory cytokines in response to target cell exposure. Our data support the further development and subsequent implementation of our 8D10F10-based biAb as an immunotherapy for HL and other CD30^+^ malignancies.

## Materials and methods

### Anti-CD30 monoclonal antibody (mAb) development

The cDNA for the extracellular amino acids (19-379) of the huCD30 sequence (GenScript) were fused to the glutathione S-transferase (GST) sequence in a pGex-4T-1 plasmid (GE Healthcare). The BL21 *E. coli* strain (NEB) was transformed and expression was induced with IPTG (Sigma). Expression was confirmed by Coomassie-stained SDS-polyacrylamide gel and Western blot analysis using an anti-GST antibody (ThermoFisher). huCD30-GST protein was purified to apparent homogeneity with Glutathione Sepharose 4B Resin (GE Healthcare) and buffer-exchanged (Millipore Amicon^®^ Ultra-4 Centrifugal Filter Unit 50 KDa cutoff) to PBS. Mouse CD30 hybridomas (clones 8D10, 10C2, 12B1, 13H1, 15B8) (all custom generated by Genscript), were derived by fusing murine Sp2/0 myeloma cells with spleen cells from a BALB/c mouse that had been immunized with purified huCD30-GST and boosted. Hybridoma lines that grew out of the selection media were maintained in DMEM media (Gibco) + 10% FBS (Gibco).

### Antibodies and reagents

Hybridoma cell lines were adapted to CD Hybridoma AGT medium (Gibco); a serum-free media (SFM), supplemented with 40 mL/L GlutaMAX (ThermoFisher). Supernatants were harvested; mAbs were purified on an rProtein A GraviTrap column (GE Healthcare). mAbs were concentrated and buffer-exchanged (Millipore) to PBS. Glycerol was added to 50% and the purified mAbs were stored at -20°C. Each mAb was quantified by densitometry using ImageJ (27) and/or by a BCA protein assay. mAb light and heavy chains were verified using a non-reducing SDS-polyacrylamide gel (Bio-Rad). mAbs were either used unlabeled or fluorescently-labelled with an Alexa Fluor 647 labeling kit (Invitrogen).

The anti-CD30 mAb clone AC10, which is the mAb component of brentuximab, was obtained from AdipoGen (unlabeled or APC-labeled). Anti-CD30 clone BerH2 (PE-labelled) was acquired from Invitrogen. Anti-CD20-PE and anti-CXCR3-Alexa Fluor 700 were purchased from BioLegend as were all isotype control antibodies. Goat anti-mouse IgG (H+L) secondary antibody labeled with Alexa Fluor 647 was purchased from ThermoFisher.

### Cell lines and cell culture

The following cell lines were obtained from ATCC. SU-DHL-1 (CD30^+^ lymphoma), RPMI 6666 (CD30^+^ HL), Raji (CD30^low^ Burkitt’s lymphoma), and HH (CD30^+^ lymphoma) were maintained in RPMI-1640 media (Sigma), supplemented with 10% FBS (20% for RPMI 6666) (Gibco). K562 (CD30^+^ CML) and 293T (CD30^-^) were maintained in DMEM media (Gibco), supplemented with 10% FBS (Gibco).

OCIAML-2 (CD30^−^ AML) are from the Ontario Cancer Institute and maintained in IMDM media (Gibco), supplemented with 10% FBS (Gibco). Karpas 299 cells (CD30^+^ NHLCL) are from Sigma and cultured in the same media as HH (above). L428 (CD30^+^ HL) are from DSMZ and cultured in X-VIVO 20 media with 5% human AB serum.

A binary CD30^+^ cell line (Raji LV30) was derived by transducing CD30^-^ Raji cells with a lentiviral vector (LV) construct containing the cDNA for huCD30. The sequence for full-length, huCD30 (UniProt) was codon-optimized (Genscript) and subcloned into the pDY LV vector (28). A high-titer LV prep was used to transduce Raji cells, which were sorted by fluorescence-assisted cell sorting (FACS) to enrich for the CD30^+^ fraction. A clonal population was developed by limiting-dilution followed by flow cytometry (FCM)-based screening to select a CD30^high^ Raji cell clone (data not shown).

Human donor mononuclear cells (MNCs) were isolated by density gradient centrifugation (StemCell Technologies) from de-identified and discarded leukocyte reduction system (LRS) cones or apheresis products or from peripheral blood (StemCell Technologies). MNCs were used fresh or frozen and stored in the vapor phase of liquid nitrogen (50% X-VIVO 20 media (Lonza), 40% human AB serum (Gemini Bio-Products), and 10% DMSO (Sigma)) and thawed prior to use. T cells were activated and expanded with CD3/CD28 Dynabeads (ThermoFisher) or 20 ng/mL anti-CD3 antibody (OKT3; BioXCell) and 100 IU/mL interleukin-2 (IL-2) (Sigma or NIH/NCI BRB Preclinical Repository) in complete media: X-VIVO 20 media (Lonza) with 5% human AB serum (Gemini Bio-Products).

### Binding studies: ELISA and flow cytometry (FCM)

Half-area microtiter plates (Corning) were coated with huCD30-GST protein at 10 ug/mL in PBS for 1 hour at RT or ON at 4°C. After blocking: 5% bovine serum albumin (BSA) solution in PBS (1 hour, RT), purified mAbs were incubated in serial dilutions with the CD30-GST protein at RT. After 1 hour, the wells were washed with PBS-Tween (0.05%) and the bound antibodies were detected by incubating the cells with an alkaline phosphatase-linked anti-mouse IgG probe (Cell Signaling Technology) at RT for 1 hour. The excess probe was washed off and the plate was developed with 1-step pNPP solution (ThermoFisher). The reaction was terminated with the addition of 0.75M NaOH after 30 minutes. The optical density at 405 nm was determined using a Varioskan LUX plate reader (ThermoFisher).

Cell lines were stained for 30 minutes at 4°C with purified anti-CD30 mAbs (all at 5 μg/mL), followed by Alexa Fluor 647-labeled secondary antibody (1:2000) for 30 minutes. Alternatively, cell lines and T cells were stained in FACs buffer (2% FBS, 1 mM EDTA, 0.02% NaN_3_ in PBS) with 8D10 or 10C2 biAbs (5 μg/mL), followed by staining with Alexa Fluor 647-labeled secondary antibody as above. Cells were washed and fixed with 1% PFA. Stained cells were analyzed on a BD LSR Fortessa X-20 flow cytometer (BD Biosciences) with acquisition of least 1x10^4^ events. Data were analyzed using FlowJo v10 software (FlowJo LLC).

### mAb sequencing

The variable light (V_L_) and heavy (V_H_) chains were sequenced. RNA was extracted from each hybridoma cell line using TRIzol (Invitrogen) and chloroform (Sigma) and converted to cDNA. V_L_ and V_H_ chains were amplified by PCR using mouse-specific primers and subcloned into a shuttle vector (pBlueScript II SK(+)) for sequencing. Plasmids were sequenced by Sanger sequencing (Retrogen). The similarities of the predicted amino acid sequences of the five novel anti-CD30 mAbs and AC10 was determined *via* ClustalOmega (29). Mass spectrometry (MS) was used to perform bottom-up analysis of the mAb amino acid sequences to compare to the DNA results. The mAbs were reduced, cysteines were alkylated, and the protein was digested by Lys-C and trypsin. Peptides were analyzed by high mass accuracy mass spectrometry (LC-MS/MS, Orpitrap Fusion Lumos) by the Center for Biomedical Mass Spectrometry Research at MCW. Data was searched against the amino acid sequences determined by DNA sequencing.

### Binding affinity analyses

Surface plasmon resonance (SPR) measurements were performed at 25°C using a BIAcore 3000 instrument (GE Healthcare). CM5 and Protein G chips, surfactant P20, and amine coupling kits were obtained from GE Healthcare. Recombinant huCD30 (R&D) at 10 ng/ul was immobilized on a CM5 sensor chip by amine coupling. Unreacted groups were blocked with ethanolamine. Our purified anti-CD30 mAbs and clone AC10 were prepared in running buffer (10 mM Hepes pH 7.4, 150mM NaCl, 0.005% (v/v) P20) with 0.1% BSA in serial 1:2 dilutions from 20 nM. mAbs were injected in a volume of 150 ul over the flow cells at a flow rate of 30 ul/min. After 5 min, the antibody-containing solution was replaced with running buffer containing BSA and the complexes were allowed to dissociate for 10 min. The CM5 sensor chip was regenerated with a one-minute injection of 20 mM glycine, 1M NaCl, pH 2.5, at a flow rate of 10 ul/min; the surface was allowed to re-equilibrate in running buffer for 3 min prior to subsequent injections.

The reverse analysis was also carried out. Here, mAbs 8D10, 10C2, AC10, and isotype control (each at 5 ng/ul) were immobilized to a Protein G sensor chip at an approximate density of 200 RU in running buffer (described above) at a flow rate of 10 ul/min. Recombinant huCD30 (8D10 and 10C2 flow cells: 0-2500 nM; AC10 flow cell: 0.1-25 nM) was injected in running buffer for 2 min at 40 ul/min with a dissociation time of 3 min. The Protein G chip was regenerated with a 1 min injection of 20 mM glycine pH 2.5, 150 mM NaCl.

Concentration curves were fitted to a 1:1 Langmuir binding model using the BIAevaluation software package (version 4.1.1; GE Healthcare) from which kinetic rate constants and equilibrium constants were calculated.

### Epitope cluster mapping and linear epitope binding

HuCD30 epitope cluster mapping was assessed by an FCM-based competitive binding assay. SU-DHL-1 cells were first blocked with unlabeled anti-CD30 mAbs (2.5 ug per 100 uL) for 1 hour at 4°C. The cells were washed and then incubated with Alexa Fluor 647-labeled 8D10, 10C2, 12B1, 13H1, or 15B8, APC-labeled AC10, or PE-labeled BerH2 for 30 minutes at 4°C. Excess antibody was washed from the cells, and fluorescence was determined using a BD LSR Fortessa X-20 flow cytometer (BD Biosciences) with acquisition of least 2x10^4^ events. Data were analyzed using FlowJo v10 software (FlowJo LLC).

Actual anti-huCD30 linear epitope binding was assessed by PEPperPRINT, GmbH (Heidelberg). The huCD30-GST sequence was converted into linear 15 amino acid peptides with a peptide-peptide overlap of 14 amino acids. The resulting CD30 peptide microarrays contained 586 different peptides printed in duplicate (1172 spots) and were framed by additional HA (YPYDVPDYAG, 86 spots) control peptides. MAbs 8D10F10 (see below) and AC10 were analyzed at 1 ug/mL, 10 ug/mL, and 100 ug/mL in incubation buffer (PBS, pH 7.4 with 0.05% Tween 20 and 10% Rockland blocking buffer MB-070). Microarray read-outs were obtained with an Innopsys InnoScan 710-IR Microarray scanner. Microarray image analysis was done with PepSlide^®^ Analyzer.

### Generation of biAbs and arming of T cells

BiAbs with OKT3 and either 8D10, 10C2, or AC10 were prepared by chemical heteroconjugation as previously described (30). OKT3 was cross-linked with Traut’s reagent (2-iminothiolane HCl; Pierce) and the anti-CD30 mAbs were cross-linked with sulphosuccinimidyl 4-(N-maleimidomethyl) cyclohexane-1-carboxylate (Sulpho-SMCC). Cross-linked mAbs were desalted on PD-10 columns (Pharmacia) to remove unbound cross-linker. The cross-linked OKT3 and anti-CD30 Abs were then heteroconjugated overnight. Heteroconjugated product was analyzed by non-reducing SDS–PAGE (4–20% gradient; Lonza). Bands were quantified by densitometry using Quantity One software (Bio-Rad Laboratories).

T cells were cultured as described above for at least 5 days prior to arming. The activated T cells were armed with 8D10, 10C2, or AC10 biAb at 100 ng per 1x10^6^ cells, unless otherwise specified, for 1 hour in complete media at 4°C prior to use or they were armed and cryopreserved as described above.

### Cell conjugation assay

8D10 or 10C2 biAb-armed T cells were labelled with Alexa Fluor 700 using an anti-CXCR3 antibody. Target cells (Raji and Raji LV30) were labelled with phycoerythrin (PE) using an anti-CD20 antibody. All cells were washed, resuspended in FACS buffer, and mixed at a 1:1 ratio of tumor cells to target cells. Unarmed T cells were included as a control. Cells were co-incubated for 1 hour at 4°C and analyzed immediately on a BD LSR Fortessa X-20 flow cytometer. At least 2x10^4^ events were collected for each sample. Data were analyzed using FlowJo v10 software. Two-colored particles were scored as positive cell conjugations.

### Cytokine profiling of biAb-armed T cell and tumor cell co-cultures

BiAb-armed T cells were incubated at a 2:1 effector-to-target ratio with tumor cells (2x10^5^ cells per condition). Samples were diluted 1:2, and cytokine concentrations determined using standard curves. For unarmed T cells, 8D10 biAb-armed T cells, and 10C2 biAb-armed T cells incubated with Raji, Raji LV30, SU-DHL-1, RPMI 6666, and OCIAML2 cells the multiplex panel included CD40L, EGF, Eotaxin, FGF-β, Flt-3 ligand, Fractalkine, G-CSF, GM-CSF, Granzyme B, GRO-α, GRO-β, IFN-α, IFN-β, IFN-γ, IL-10, IL-12 p70, IL-13, IL-15, IL-17A, IL-17E, IL-1α, IL-1β, IL-2, IL-3, IL-33, IL-4, IL-5, IL-6, IL-7, IL-8, IL-1ra, IP-10, MCP-1, MIP-1α, MIP-1β, MIP-3α, MIP-3β, PD-L1, PDGF-AA, PDGF-AB/BB, RANTES, TGF-α, TNF-α, TRAIL, and VEGF (Magnetic Luminex Performance Assay XL Kit, R&D Systems). For unarmed T cells and 8D10 biAb-armed T cells incubated with Raji and HH cells the multiplex panel included GM-CSF, IFN-α2, IFN-γ, IL-2, IL-6, IL-10, IL-12 (p40), IL-12 (p70), and TNF-α (custom MILLIPLEX Human Cytokine/Chemokine/Growth Factor Panel A-Immunology Multiplex Assay, Sigma).

### BiAb-dependent cytotoxicity

Tumor cell lines were labeled with ^51^Cr (Perkin Elmer) for 2 hours at 37°C; then washed twice with 2% FBS in PBS and once with X-VIVO 20 media containing 5% human AB serum. T cells were plated with the target cells in triplicate at varying effector-to-target (E:T) ratios and incubated for 4 hours at 37°C. Following incubation, 30 uL aliquots of supernatant were collected into 96-well LumaPlates (Perkin Elmer) and read using a TopCount NXT Scintillation Counter (Packard) or Microbeta A2450-0020 Plate Reader (Perkin Elmer). Spontaneous release was measured by incubation of target cells with media alone. Maximum release was achieved by lysis of target cells with a 0.05% Triton-X solution. Specific lysis was calculated using the formula: % specific lysis = [(experimental release - spontaneous release)/(maximum release - spontaneous release)] x 100. Triplicate values were averaged prior to calculation of the specific lysis; data from multiple experiments were averaged.

### Subcloning hybridoma 8D10

Hybridoma 8D10 was subcloned by limiting dilution into a 96-well plate at a cell density that predicted only 1 hybridoma per every 3-4 wells. Wells were scanned the same day for those containing more than 1 cell, which were eliminated. Five single cell-derived subclones of 8D10 were isolated and examined for mAb production. Clone 8D10F10 was selected for further study. Anti-CD30 mAb 8D10F10 was purified on an rProtein A GraviTrap column. Clone 8D10F10 was sent to IDEXX BioAnalytics, Inc., for analyses and was found to be negative by PCR testing for all 25 mouse viruses examined by their IMPACT I array (data not shown).

### mAb specificity analyses

Antibody 8D10F10 was tested in a Membrane Proteome Array analysis (Integral Molecular) against a library of over 6000 human membrane proteins including 94% of all single-pass, multi-pass, and GPI-anchored proteins. Membrane proteins were expressed from plasmids transfected into HEK-293T cells. The cells were matrixed by pooling individual columns and rows of each plate. Ten percent goat serum was added in the blocking buffer. The primary antibody (8D10F10) was incubated with cells for 60 minutes at RT. Binding was determined by FCM using an Intellicyt iQue and a fluorescently labeled secondary antibody incubated for 30 minutes at RT. The optimal 8D10F10 antibody screening concentration was found to be 1.25 ug/mL; this concentration was chosen based on a joint assessment of binding strength and background signals (data not shown). Targets were identified by detecting ligand binding to overlapping column and row pools. Each individual membrane protein target was assigned values corresponding to the binding values of their unique row and column pools, and targets demonstrating binding signal >3 standard deviations above background in both wells were selected for downstream validation experiments. The resulting paired binding values were subsequently normalized and transformed to give a single numerical value for binding of the test ligand against each target protein. Non-specific fluorescence was determined to be any value below 3 standard deviations of the mean background value. Identified targets were validated in secondary screens that involved transfections with plasmids encoding the specific test cDNAs and serial dilutions of 8D10F10 followed by FCM analyses as above.

### *In vivo* analyses

Under a protocol approved by the Animal Use Committee of MCW (#AUA00005801), NSG mice (Jackson Labs) were injected intradermally with 4x10^6^ HH cells. Beginning on day 10, when tumor volume reached ~100mm^3^, the mice received 6 intravenous injections of either PBS, 20x10^6^ unarmed T cells, or 20x10^6^ 8D10 biAb-armed T cells (armed as above, cryopreserved and thawed) every 3 to 4 days. Tumor size (length x width^2^/2) and ulceration status were determined every 3 to 4 days. Mice with ulcerated tumors were euthanized per the protocol requirement. All mice were euthanized at day 28 when most control mice had ulcerated tumors.

### Statistical analysis

All statistical analyses were done using Graph-Pad Prism version 8 for Mac (GraphPad Software, San Diego). Results from conjugation assays were compared by one-way ANOVA with Tukey’s multiple comparison test. Luminex cytokine data and T cell cytokine release data were compared by two-way ANOVA with Dunnett’s multiple comparisons test. Cytotoxicity data were compared by two-way ANOVA with Tukey’s multiple comparison test. *In vivo* data were compared by a log-rank (Mantel-Cox) test. Further statistical tests are described in figure legends. P values of ≤ 0.05 were considered statistically significant; Non-significant, p > 0.05; *, p < 0.05; **, p < 0.01; ***, p < 0.001.

## Results

### Production and analysis of novel mAbs against the huCD30 extracellular domain

**Figure 1:**
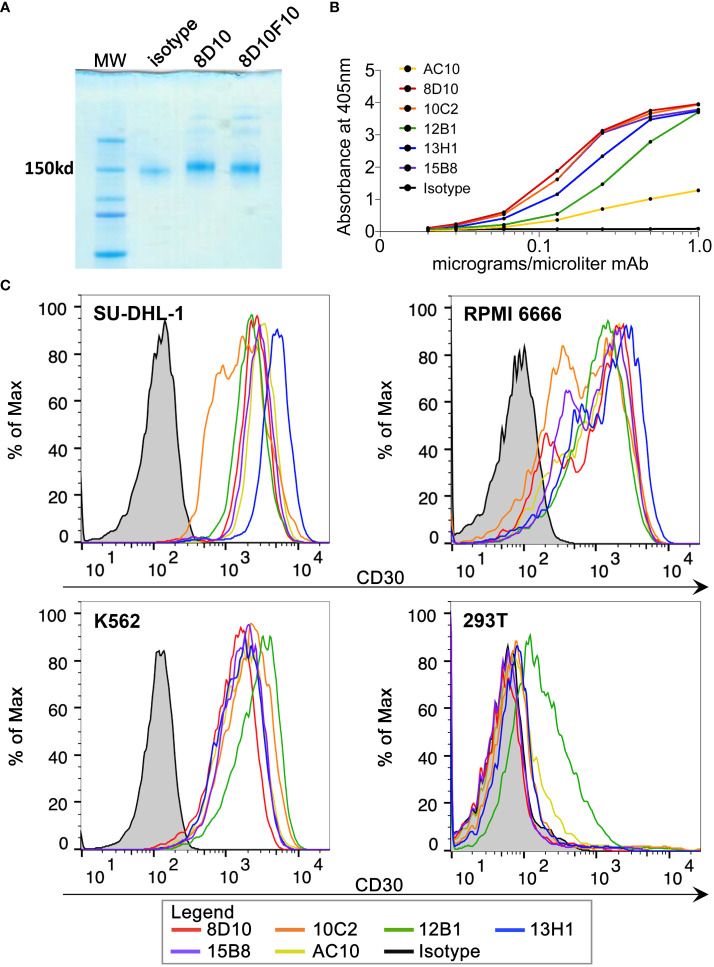
Purification of novel anti-CD30 mAbs and binding to huCD30. **(A)** Coomassie Blue-stained gel following non-reducing PAGE of purified 8D10 and 8D10F10 (subclone for preparation of a clinical trial MCB). **(B)** Binding of various dilutions of our novel mAbs to huCD30-GST protein was assessed by ELISA. **(C)** FCM analyses were carried out for all five novel CD30 mAbs and AC10. Representative FCM histograms are shown.

Five hybridoma cell lines were produced following inoculation of mice with huCD30-GST. Hybridomas 8D10, 10C2, and 13H1 were found to secrete human immunoglobulin G2a (IgG2a) with kappa light chains, while hybridomas 12B1 and 15B8 were found to produce IgG2b with kappa light chains (as assessed by Genscript). MAbs were purified from hybridoma supernatants by protein A chromatography. **Figure 1A** shows the Coomassie-stained results of non-reducing SDS-PAGE with mAb 8D10 compared to an isotype control mAb.

Binding of each mAb from the five hybridoma cell lines (8D10, 10C2, 12B1, 13H1, 15B8) to huCD30 was first assessed by ELISA (**Figure 1B**). AC10 (the antibody component of brentuximab) was also included in these analyses. All 6 anti-CD30 mAbs demonstrated dose-dependent binding to CD30-GST. As expected, an isotype control mAb did not bind. Our 5 mAbs against CD30-GST produced a stronger signal in this ELISA than AC10 at the same concentration (**Figure 1B**). This may be due to differences in affinity or concentration or the fact that CD30-GST was the exact immunogen used for development of our mAbs whereas AC10 was raised by immunization of mice with YT cells (a CD30^+^ lymphoma cell line) (31).

An assessment by FCM was carried out to analyze binding of our anti-CD30 mAbs to their ligand in its native context (**Figure 1C**). SU-DHL-1 and RPMI 6666 are CD30^+^ lymphoma cell lines. K562, a CML cell line, also expresses CD30. 8D10, 10C2, 12B1, 13H1, and 15B8 all bound to the three CD30^+^ cell lines with a similar mean fluorescence intensity (MFI) for each cell line. AC10 also stained these cell lines with a similar MFI. Only 12B1 showed appreciable binding to CD30^-^ 293T cells. To further prove antigen-specific binding, the huCD30 cDNA sequence was transduced into the Raji cell line using a recombinant LV we constructed, and a clonal CD30^+^ population was derived (Raji LV30). Staining of non-transduced (NT) Raji cells with our 5 mAbs and AC10 revealed a low to moderate intensity of binding (**Supplemental Figure 1**). This is consistent with at least one previous report that Raji cells express low levels of CD30, despite the fact that it is often cited and used as a CD30 negative cell line (32). For all the mAbs tested, binding to Raji LV30 cells was significantly higher than to NT Raji cells, demonstrating specificity of all 5 novel mAbs (and AC10) for the expressed CD30 protein (**Supplemental Figure 1**).

### mAb binding affinities

Affinity for huCD30 was assessed by SPR analyses. Our mAbs all bound to rhCD30 protein immobilized as the ligand on a CM5 sensor chip with affinities in the nM range, as expected for antibody-antigen interactions (**Table 1**). These results were comparable to the binding affinity determined for AC10 (**Table 1**). This data is consistent with previous studies of the binding affinity of brentuximab, which have reported a K_d_ between 0.2 - 2 nM (33–35). Binding curves and Langmuir fits are shown in **Supplemental Figure 2**.

**Table 1:** Affinity constants for the interaction of CD30 mAbs with immobilized huCD30 protein.

Antibody	K_A_ (M^-1^)	k_a_ (Ms^-1^)	K_D_ (nM)	k_d_ (s^-1^)
**8D10**	16.9x10^9^	10.1x10^4^	0.0592	0.6x10^-5^
**10C2**	1.65x10^9^	30.7x10^4^	0.607	18.6x10^-5^
**12B1**	5.02x10^9^	6.13x10^4^	0.199	1.22x10^-5^
**13H1**	6.7x10^9^	70.8x10^4^	0.149	10.6x10^-5^
**15B8**	1.02x10^9^	50.4x10^4^	0.978	49.3x10^-5^
**AC10**	6.77x10^9^	130x10^4^	0.148	19.2x10^-5^

To assess any differences when the mAbs were used as the ligand rather than as the analyte, 8D10, 10C2, and AC10 were also tested by immobilization on a Protein G sensor chip. Here, rhCD30 was the analyte (**Supplemental Table 1**). Somewhat surprisingly, in this context 8D10 and 10C2 binding affinity values were reduced to the μM range. AC10 still bound rhCD30 with nM affinity, though the off-rate was significantly increased indicating that immobilization of anti-CD30 antibodies themselves may not be the best method for assessment.

### Sequence analysis and epitope studies

**Figure 2:**
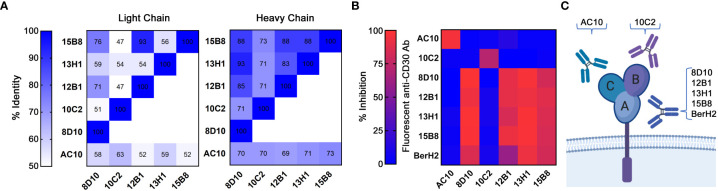
Categorization of mAbs by sequence and cluster binding. **(A)** V_L_ and V_H_ protein sequences were compared across all 5 novel mAbs and AC10. Levels of sequence similarity range from white (low) to blue (high). Numbering indicates the sequence identity as a percentage. **(B)** Cluster mapping *via* competitive binding. Fluorescently labelled mAbs were incubated with CD30^+^ SU-DHL-1 cells blocked with excess unlabeled mAbs. FCM analysis was then performed. High inhibition (red) indicates a shared cluster and low inhibition (blue) indicates distinct clusters. Results represent the means of 3 independent experiments (n=3). **(C)** A depiction of the three known clusters of mAb binding to CD30.

To confirm the uniqueness of our novel anti-CD30 mAbs, the V_L_ and V_H_ chain antibody sequences were determined (**Supplemental Table 2**; with a distance-based sequence similarity tree in **Supplemental Figure 3**). The sequence of AC10 has been previously reported (36). Comparisons revealed that each mAb is unique (**Figure 2A**). Predicted amino acid sequences ranged from 50%-94% similar for the V_L_ chains, and 68%-90% similar for the V_H_ chains. No V_L_ or V_H_ chain in our cadre of novel anti-CD30 mAbs was found to be >73% similar to the AC10 sequence. Subsequently, MS-based protein sequencing matched the predicted amino acid sequences (data not shown).

Currently characterized anti-CD30 mAbs recognize one of three serologically defined clusters (A, B, or C) (37). AC10 binds to cluster C, while a second commercially available antibody, BerH2, binds to cluster A (37). We examined the ability of unlabeled mAbs to inhibit the binding of fluorescently-labelled mAbs to CD30^+^ SU-DHL-1 cells in order to assign cluster groups (**Figure 2B**; **Supplemental Figure 4**). A known cluster group B antibody could not be identified from this analysis. Binding of AC10 was not inhibited by any of our mAbs, indicating that none of our mAbs bind to cluster C. Binding of 8D10, 12B1, 13H1, 15B8 and BerH2 was inhibited by unlabeled 8D10, 12B1, 13H1, and 15B8, indicating that these antibodies share a common cluster. Since BerH2 is known to belong to cluster A, we assigned these 4 of our 5 antibodies to this cluster. Our fifth antibody, 10C2, did not inhibit binding of any other antibody tested, thus we conclude that it binds to cluster B, or to a novel cluster (**Figure 2C**). For further studies, we selected one antibody from each cluster group: 8D10, which had the highest affinity of all our cluster A antibodies, and 10C2, which is unique in both sequence and epitope cluster.

To define the epitopes on human CD30 that our lead antibody (subclone 8D10F10; see below) recognizes, and to compare this to results with AC10, we tested binding to linear CD30 peptide microarrays. Incubations were performed at different concentrations of input mouse antibodies. For 8D10F10, moderate-to-very strong antibody responses against epitope-like spot patterns formed by adjacent peptides with the consensus motifs EEKYEE, DFMLYD, and CEPDYYLDE were found at high signal-to-noise ratios. In fact, the proposed epitope CEPDYYLDE was found twice in the antigen (**Supplemental Figure 5**). In contrast for AC10, moderate-to-strong antibody responses against epitope-like spot patterns formed by adjacent peptides with the consensus motifs YWKIKGLVQPTR, LYERDEGDKWRNK, TQQCPQRPTDCR, GTRLAQEAASK, and FKKRIEAIPQIDKYL were observed at high signal-to-noise ratios (**Supplemental Figure 5**). These response profiles were both observed at similar intensity levels; no common antibody responses were found. This further confirms that mAbs 8D10F10 and AC10 bind to different regions of human CD30.

### Human T cell-to-tumor cell conjugation

**Figure 3:**
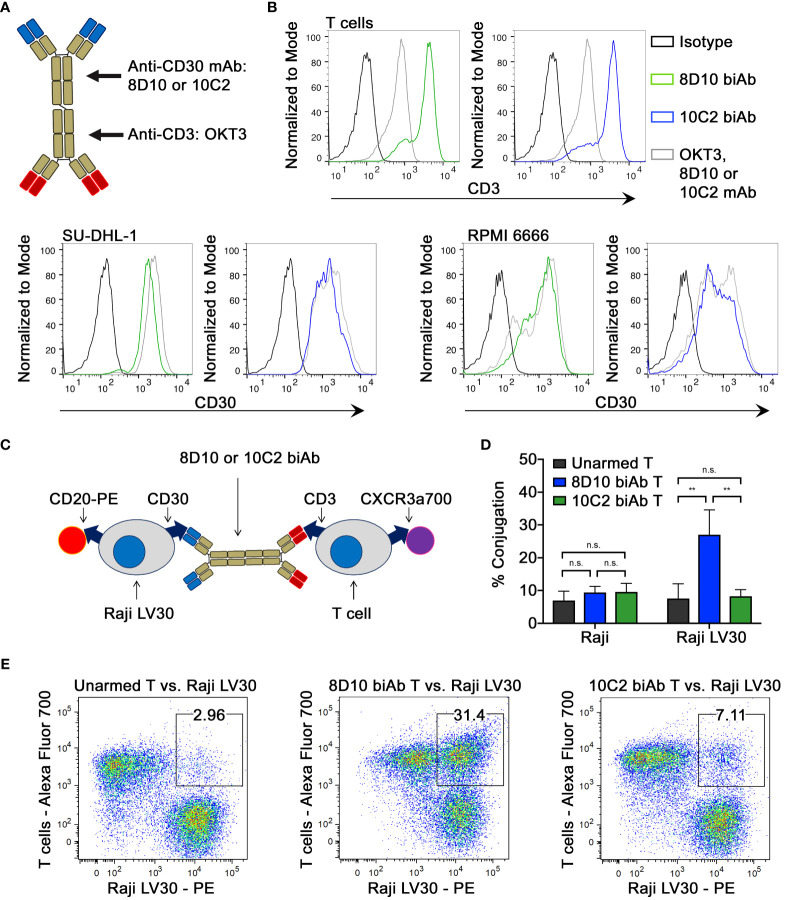
Assessment of biAb binding capabilities. **(A)** BiAbs capable of binding to CD3 and CD30 were developed by heteroconjugation of mAb 8D10 or 10C2 with OKT3. **(B)** The ability of biAbs 8D10 and 10C2 to bind to both CD3 and CD30 post-conjugation was determined by FCM. Binding of unconjugated 8D10, 10C2, and OKT3 mAbs and an isotype control is shown for reference. **(C)** A two-color FCM assay was developed to determine T cell-Raji LV30 conjugate formation in the presence of biAbs. **(D)** Assessment of percent conjugation of Raji and Raji LV30 cells with unarmed or biAb-armed T cells was measured by detection of two-color events and plotted as a percentage of all events. Results represent means ± SD of 3 independent experiments (n = 3). Representative FCM plots from this assay are shown in **(E)**. n.s, not significant, ** = p < 0.01 between the indicated groups, calculated using a one-way ANOVA with Tukey’s multiple comparisons test.

Heteroconjugation of equimolar concentrations OKT3 and either 8D10 or 10C2 mAb was carried out (**Figure 3A**; **Supplemental Figure 6**). To establish that the ability to bind to both CD3 and CD30 was maintained in the biAb products, CD3^+^ T cells, CD30^+^ SU-DHL-1, and RPMI 6666 cells were stained with 8D10 or 10C2 mAb and biAbs and analyzed by FCM. Strong binding to T cells, SU-DHL-1, and RPMI 6666 cells was observed by both biAbs (**Figure 3B**). Both biAbs bound with a similar MFI as the original mAbs, confirming their ability to bind to both proteins of interest even after heteroconjugation.

An essential component of biAb function is the capacity to bind both target proteins simultaneously. We assessed T cell-to-tumor cell conjugation using a two-color FCM approach (**Figure 3C**). Tumor cells and armed T cells, each stained with different fluorophores, were co-cultured prior to analysis. Two-color conjugates were scored (**Figures 3D, E**). As expected, there was little conjugation between non-CD30 expressing (NT) Raji cells and unarmed or armed T cells. In comparison, T cells armed with the 8D10 biAb showed significantly greater levels of conjugation to Raji LV30 cells. Unexpectedly, 10C2 biAb-armed T cells demonstrated no significant conjugation with Raji LV30 cells (**Figures 3D, E**). This indicates a weaker ability of the 10C2 biAb to mediate cellular heteroconjugations due to spatial restrictions or formation of less stable pairings that were too fragile to be evaluated by FCM.

### *In vitro* cytokine production

**Figure 4:**
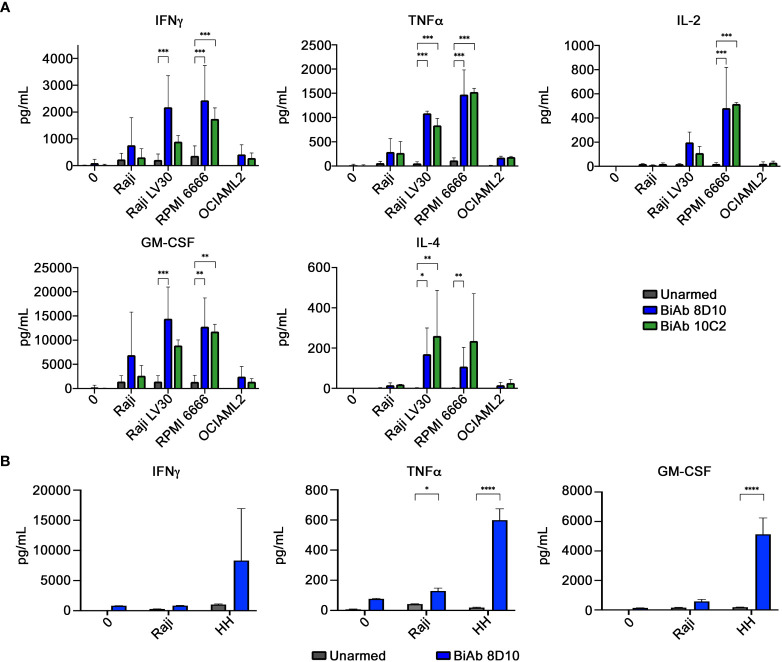
Cytokine production by biAb-armed T cells after co-culture with tumor cells. **(A)** Co-culture conditions included no target (0), a CD30^low^ cell line (Raji), CD30^+^ cell lines (Raji LV30 and RPMI 6666), and a CD30^−^ cell line (OCIAML2). Production of IFN-γ, TNF-α, IL-2, GM-CSF, and IL-4 was assessed. **(B)** Co-culture conditions included no target (0), a CD30^low^ cell line (Raji), and a CD30^+^ cell line (HH). Results represent means ± SD of 3 independent experiments (n = 3). * = p < 0.05, ** = p < 0.01, *** = p < 0.001 between the indicated groups, calculated using a two-way ANOVA with Dunnett’s multiple comparisons test.

We evaluated the ability of our biAb-armed human T cells to produce cytokines when challenged with CD30-expressing tumor cell lines at an E:T ratio of 2:1. T cells from three independent donors were used. Significant increases in production of GM-CSF, Granzyme B, IFN-γ, IL-2, IL-4, IL-5, IL-10, IL-13, MIP-1α, MIP-1β, and TNF-α were observed in supernatants collected from 8D10 or 10C2 biAb-armed T cells co-cultured with CD30-expressing tumor cells (Raji LV30 and RPMI 6666) in comparison to co-culture with unarmed T cells (**Figure 4A**; **Supplemental Figure 7**). Much lower levels of cytokines were detected when armed T cells were incubated with CD30^−^ cell lines (Raji and OCIAML2). Production of other cytokines was low across all samples; often below the limit of detection (data not shown). Interestingly, despite the negligible increase of T-cell-to-tumor cell conjugation in the presence of 10C2 biAb in the FCM assay (**Figure 3**), this biAb triggered production of some cytokines *in vitro*. However, in comparison to cell conjugation with 8D10 biAb-armed T cells, 10C2 biAb-armed T cells trend towards less IFN-γ, GM-CSF, IL-5, IL-10, MIP-1β, and Granzyme B.

### *In vitro* cytotoxicity

**Figure 5:**
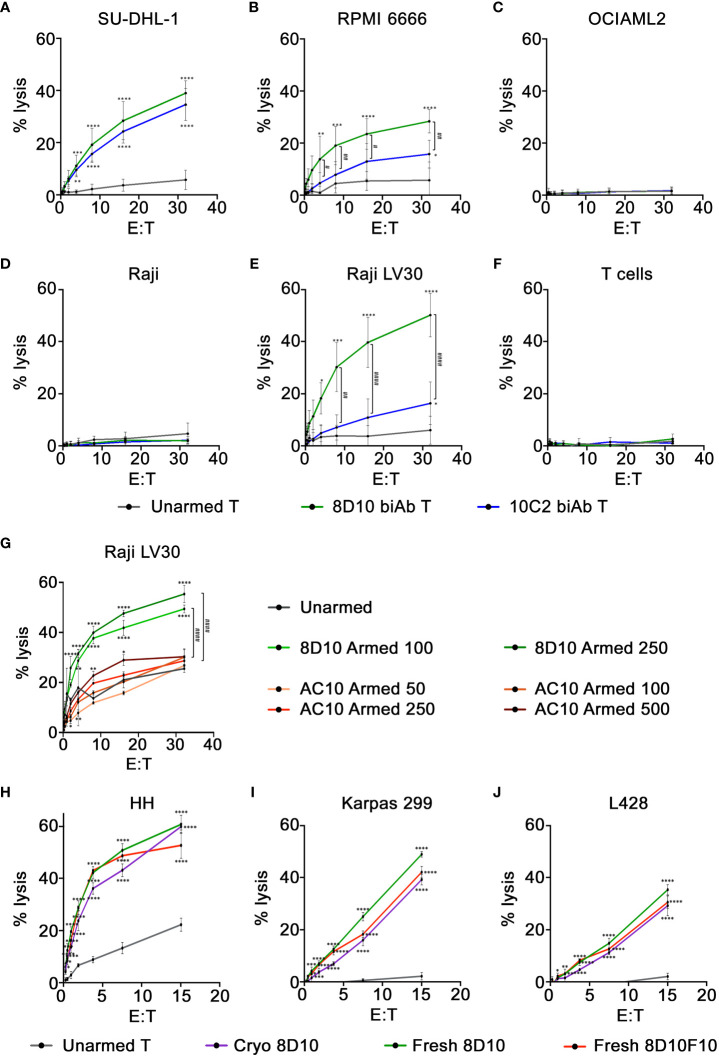
Cytotoxicity of CD30/CD3 biAb-armed T cells. **(A–F)** Cytotoxicity of 8D10 & 10C2 biAb-armed T cells against CD30^−^ and CD30^+^ tumor cells was assessed using a standard 4-hour ^51^Cr release assay. Lysis of two tumor cell lines with endogenous CD30 expression, **(A)** SU-DHL-1 and **(B)** RPMI 6666, was measured, in addition to **(C)** OCIAML2, a CD30^−^ cell line, **(D)** NT Raji, a CD30^low^ cell line, and **(E)** Raji LV30 cells, which are CD30^high^. **(F)** Activated T cells, which express low levels of CD30, were also assessed for lysis. Cytotoxicity of unarmed, 8D10, and AC10 biAb-armed T cells was compared at 24 hours against Raji LV30 cells **(G)** after T cells were armed with varying levels (50-500ng/million cells) of biAb. Cytotoxicity of 8D10 and 8D10F10 biAb-armed human T cells used fresh or after cryopreservation against CD30^+^ cell lines: **(H)** HH, **(I)** Karpas, and **(J)** L428 was ascertained. Results represent means ± SD of 3-4 independent experiments per cell line (n = 3-4). * = p < 0.05, ** = p < 0.01, *** = p < 0.001, **** = p < 0.0001 between biAb-armed T cells and unarmed T cells. # = p < 0.05, ## = p < 0.01, #### = p < 0.0001 between 8D10 and 10C2 biAb-armed T cells **(A–F)** and between 8D10 and AC10 biAb-armed T cells **(G)**. Statistical significance was calculated using a two-way ANOVA with Tukey’s multiple comparisons test.

To evaluate the *in vitro* cytotoxicity of 8D10 and 10C2 biAb-armed T cells against multiple CD30^+^ tumor cells of different origins, biAb-armed T cells were co-cultured with ^51^Cr-loaded tumor cells. An arming dose of 100 ng biAb per 1x10^6^ cells was found to be optimal (data not shown). Specific lysis at E:T ratios from 32:1 to 0.25:1 was determined by evaluation of the ^51^Cr signal in the cell culture supernatants. Significantly higher lysis was observed against the CD30^+^ cell lines SU-DHL-1, RPMI 6666, and Raji LV30 by biAb-armed T cells compared to that obtained with the unarmed T cells (**Figures 5A, B, E**). Little to no cytotoxicity by either 8D10 or 10C2 biAb-armed T cells was observed against the CD30^−^ cell line OCIAML2 or NT Raji cells (**Figures 5C, D**). The 10C2 biAb-armed T cells had equal activity as the 8D10 biAb-armed T cells against SU-DHL-1 cells; however, consistent with the heteroconjugation assays above, the 10C2 biAb was significantly outperformed by the 8D10 biAb-armed T cells against RPMI 6666 and Raji LV30 cells. Based on the 8D10 biAb engendering the highest cytolytic activity against the widest range of CD30^+^ target cells it was selected for future preclinical development assessments.

Activated T cells can express CD30 on their cell surface. This could result in fratricide, causing elimination of the immunotherapeutic product. To test the cytotoxicity of our 8D10 and 10C2 biAb-armed T cells against T cells themselves, activated T cells were loaded with ^51^Cr and used as target cells in the cytotoxicity assay. These cells were assessed by FCM and found to express low levels of CD30 on a sub-population of T cells (data not shown). Neither 8D10 nor 10C2 biAb-armed T cells displayed cytotoxicity against activated T cells with CD30 expression (**Figure 5F**).

For an *in vitro* cytotoxicity comparison, we also heteroconjugated OKT3 with AC10 and armed ATCs with doses of 50–500 ng biAb per 1x10^6^ cells. In parallel, T cells were also armed with 100 and 250 ng 8D10 biAb per 1x10^6^ cells. Cytotoxicity was compared against target Raji LV30 cells at various E:T ratios (**Figure 5G**). Somewhat surprisingly, the AC10 biAb-loaded T cells were not more cytotoxic than unarmed cells to target cells in this assay even at the highest level of arming. The 8D10 biAb-armed T cells. were significantly more cytotoxic. The lack of AC10 biAb cytotoxicity could be due to the heteroconjugation process with OKT3. These results demonstrate that the incorporation of AC10 into an anti-CD30 bispecific product produced by heteroconjugation may not be warranted.

### Preclinical development assays

Hybridoma 8D10 was subcloned by limiting dilution to facilitate analyses and in preparation for formation of a Master Cell Bank (MCB). Single cell-derived subclones were isolated, expanded, and examined for mAb production. Out of 5 candidates, subclone 8D10F10 was found to secrete 10-15 mg/L anti-CD30 mAb versus 3-5 mg/L anti-CD30 mAb for the 8D10 hybridoma pool. After purification, mAb 8D10F10 migrated as expected in non-reducing SDS-PAGE (**Figure 1A**) and was found to consist of predicted heavy and light chain components (**Supplemental Figure 8A**). Next, mAbs 8D10 and 8D10F10 were conjugated to OKT3 as above (**Supplemental Figure 8B**) and activated human T cells were loaded. Cytotoxicity of these loaded human T cells against HH cells, Karpas cells, and L428 cells was confirmed to be consistent (**Figures 5H–J**). Lastly, the efficiency of CD30^+^ tumor cell cytotoxicity was compared for freshly biAb-coated activated T cells versus biAb-coated, cryopreserved and then thawed activated T cells, consistent with anticipated clinical utilization. Both freshly loaded activated T cells and loaded cryopreserved/thawed activated T cells killed a variety of CD30^+^ tumor cells with the same efficiency (**Figures 5H–J**).

**Figure 6:**
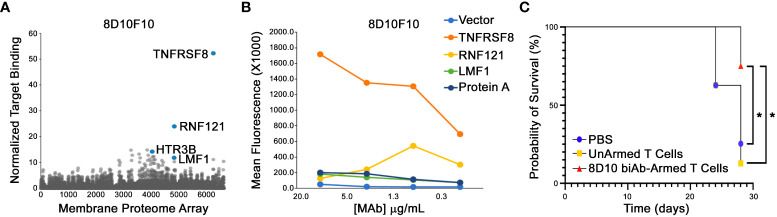
Membrane Proteome Array (MPA) analysis and *in vivo* tumor inhibition in NSG mice. **(A)** Purified 8D10F10 was tested for binding to the MPA from Integral Molecular at a previously determined optimal concentration. **(B)** Validation results. Serial ligand dilutions were incubated with newly identified targets and binding was measured by FCM. **(C)** *In vivo* survival studies. NSG mice (8 per group from 2 independent experiments) were injected with HH tumor cells (day 0) and then with PBS, unarmed, or 8D10 biAb-armed human T cells twice per week for 3 weeks from day 10. *p < 0.05 calculated using log-rank (Mantel-Cox) test.

To assess for potential off-target binding, the mAb 8D10F10 was subjected to human Membrane Proteome Array screens (Integral Molecular). The cDNAs from over 6000 human surface proteins were transfected into HEK-293T cells and represented by a unique combination of two different wells of the matrix plate. Results were determined by FCM; targets demonstrating binding signals >3 standard deviations above background in both test wells were selected for downstream secondary validation experiments. In the primary screen, 8D10F10 was found to bind strongly to TNFRSF8 (CD30) and weakly to RNF121 (ubiquitous cytoplasmic and nuclear expression), HTR3B (neuronal intracellular membrane expression), and LMF1 (endocrine and neural expression in endoplasmic reticulum) (Human Protein Atlas; www.proteinatlas.org) (**Figure 6A**) (38). In the secondary screen, cells were transfected with plasmids encoding the cDNAs from those target proteins. Antibody 8D10F10 was then added in serial dilutions. Here a target was validated if it demonstrated a binding signal in the FCM assay that was 2-fold above background at the two highest antibody concentrations tested. Only TNFRSF8 (CD30) was found to be bound by antibody 8D10F10 in the secondary screen (**Figure 6B**). Binding was also tested to TNFRSF8 (CD30), RNF121, HTR3B, and LMF1 in permeabilized cells - as some antigens may not fully track to the cell surface. As above, only TNFRSF8 (CD30) was bound here as well (data not shown).

Given the fact that HH tumor cells have been used previously in pre-clinical xenotransplant anti-cancer studies targeting huCD30 (39), and the efficiency of our 8D10 biAb-loaded T human loaded human T cells to kill HH cells *in vitro* (**Figure 5H**), we selected that cell line for our *in vivo* studies. When challenged with HH cells, 8D10 bi-Ab-loaded human T cells produced significantly more TNFα and GM-CSF (and tended to produce more IFNγ) in comparison to unarmed T cells (**Figure 4B**). Comparison of survival curves (**Figure 6C**) shows that human T cells armed with our 8D10-based bispecific significantly prolonged the lifespans of NSG mice as compared to unarmed T cells (p=0.0087) or PBS (p=0.0317). No significant difference in survival was noted between PBS and unarmed T cell treatment (p=0.6911). Actual tumor sizes are shown in **Supplemental Table 3**. No signs of metastatic disease in any of the mice were noted. Further analyses demonstrated that the unarmed T cells persisted in the blood of mice, but the 8D10 loaded cells had limited persistence in peripheral circulation (data not shown). This may be due to recognition of the immunoglobulin portion of these cells by the FcγR that we determined is present on CD45^+^ cells from the spleens of NSG mice (data not shown). Use of FcγR deficient mice or FcγR blockage could extend the persistence of 8D10 biAb-loaded T cells. and further improve outcomes in *in vivo* studies.

## Discussion

We report here the development of novel anti-CD30 biAbs that demonstrate T cell activation and cytotoxicity against CD30^+^ human tumor cell lines *in vitro* and *in vivo*. Binding constants were determined by SPR studies. Two antibodies that recognize CD30 clusters and linear epitopes, distinct from AC10, were selected for biAb development. BiAb 8D10 effectively engages the T cell receptor on T cells; both biAb 8D10 and biAb 10C2 stimulate production of T cell cytokines upon engagement of tumor cells. Furthermore, both biAbs are capable of mediating cell lysis specifically against CD30^+^ cells, while sparing CD30^−^ and CD30^low^ cells, such as activated T cells. As a result of the superior activity against multiple tumor lines, biAb 8D10 was selected as a lead candidate for *in vivo* assessment and subcloned (subclone 8D10F10) for further pre-clinical development. An MCB was prepared. A subsequent MPA analysis, screening against over 6000 human cell surface proteins, demonstrated that mAb 8D10F10 only bound to huCD30. This aligns with low off-target binding by brentuximab and should help allay safety concerns for this immunotherapy. Lastly, human T cells armed with our 8D10-based bispecific significantly prolonged the lifespans of NSG mice when they were challenged with HH tumor cells. Efficacy *in vivo* was limited due to the mouse CD45^+^ cell FcγR, however, and is a general weakness of this type of assessment.

While brentuximab has shown significant efficacy against some CD30^+^ malignancies such as HL (40, 41), the 5-year survival rate still remains unacceptable in relapsed/refractory patients (13), there are significant short- and long-term toxicities, and efficacy in CD30^+^ solid malignancies has been limited. Further, relapses/progressions following brentuximab treatment can retain CD30 expression (42). This points to a need for new therapies. As our biAbs are directed against different CD30 epitopes, they may also permit retreatment of CD30^+^ malignancies that are no longer sensitive to brentuximab due to mutations or alternative splicing; a problem that has been observed with anti-CD19 therapies (43). Two other aspects of brentuximab therapy that could be improved are short-term persistence and limited tumor penetration. Soluble biAbs and mAbs are both rapidly cleared from circulation, but pre-coating of biAbs onto activated T cells is expected to protect them from clearance, thus extending their persistence and therapeutic impact (21, 44). While many factors impact tumor penetration, including antibody affinity and antigen density, very large doses (hundreds of milligrams to grams) are routinely required in infusions for full tumor saturation, with the majority of the antibody remaining in the blood (45, 46). *Ex vivo* coating onto T cells permits our therapeutic approach to rely instead on the infiltrating abilities of T cells. BiAb-armed T cells have been shown to traffic effectively to tumors and demonstrate preferential tumor uptake and decreased clearance in comparison to unarmed T cells (47–50).

A third aspect of brentuximab therapy that may benefit from improvement is the potential for linker instability and bystander killing. Especially as, in our hands anyhow, AC10 was not a suitable heteroconjugation partner with OKT3. Brentuximab uses a cathepsin B-sensitive linker that, upon internalization by a tumor cell, is degraded to release the cytotoxic compound monomethyl auristatin E (MMAE) (51). This linker is widely used in ADC therapies because it balances plasma stability and intracellular cleavage, consequently allowing for a balance between tolerability and efficacy of the therapy (52). However, extracellular cleavage of cathepsin B has been reported, and off-target toxicities attributable to the free cytotoxic payload have been observed (53–55). Brentuximab is also thought to exert bystander killing, in which MMAE released inside a tumor cell diffuses through the cell membrane into surrounding cells regardless of their CD30 expression (56, 57). While this can be beneficial, and indeed is thought to contribute to high response rates to brentuximab even in patients whose tumors express very low levels of CD30, it can also contribute to off-target toxicity of nearby normal cells (58–60). In contrast, our biAb therapy does not include a cytotoxic compound. Furthermore, as a T cell-based therapy, biAb-armed cells have the potential to mediate effects similar to bystander killing but by a much more targeted mechanism, through antigen spreading (61). Not only were endogenous cellular, innate, and humoral responses directed against patient tumors observed when biAb-armed activated T cells were used to target multiple tumor antigens (49, 62–66), but there was also evidence for tumor antigen epitope spreading and generalized antigen spread (64).

Pre-coating T cells with biAb offers several other advantages over direct infusion of soluble biAb. Direct infusions require that the biAb independently finds both the tumor and the effector cell, which cannot then be exhausted despite residence in the tumor microenvironment (TME). The biAb surface density on immune effector cells must also reach high enough concentration to elicit anti-tumor function. In contrast, we can coat the biAb directly on a patient’s effector cells at optimal dosing. This also provides the future opportunity to maximize efficacy pre-infusion by selecting specific T cell subsets, by modifying the T cells, or by applying additional biAbs directed to other targets to both overcome tumor heterogeneity or an immunosuppressive TME. This pre-arming strategy may also decrease the risk of dose-limiting toxicities, since any unconjugated CD3 present in the biAb product will be washed away after arming and will not be freely available to bind to Fc-expressing cells *in vivo*, cause CRS, or mediate off-target effects (21, 67, 68). Additionally, the absolute amount of biAb required for an *ex vivo* arming approach is much lower (<1/30,000 of the dose given IV) than that required for direct injection, dramatically reducing production needs per patient. Lastly, our intermittent outpatient infusions of biAb-coated autologous T cells demonstrate extended persistence in circulation in contrast to small bispecifics like blinatumomab, which are rapidly cleared (62, 69). The transient nature of such bispecifics such as blinatumomab requires patients be tethered to continuous IV infusions for 28 days at a time. Continuous IV infusions can negatively impact quality of life (QOL), which is an essential component of high-quality oncology care and influences patient/family medical decision making (70, 71). Additionally, interruption of these infusions often requires inpatient readmission for monitoring of cytokine release syndrome (CRS) or immune effector cell–associated neurotoxicity syndrome (ICANS) upon reinitiating. These frequent readmissions not only reflect significant toxicity risk but also can negatively impact health-related QOL.

Previous work in the development of CD30 biAbs also includes a CD30-CD16 biAb, which targets NK cells rather than T cells (72, 73). This biAb has been studied in various forms preclinically and clinically with promising results (72, 73). Yet, isolated engagement and activation of NK cells remains a relatively unproven approach for the treatment of malignancies. For example, CD56^bright^ NK cells are the predominant tissue resident NK cell and, while excellent at production of cytokines, have poor direct cytotoxic effects. This can be augmented by IL-15 or other cytokine activation but introduces an additional hurdle not found in utilization of T cells (74). Further, while CD16 (low affinity IgG Fc receptor III) is the most potent and most studied activating receptor for NK cells for antibody-mediated activation, CD16 is easily cleaved from NK cell surface by ADAM17 (75) and attempts to inhibit this to augment NK cell activation have not yet been clinically successful (NCT02141451). NK cells also have a wide array of inhibitory receptors such as KIRs or CD94 that counteract the effects of the activation receptor repertoire (76). These remain far less investigated than activation counterparts on T cells and consequently how to prevent their regulation of anticancer augmentation attempts is limited. Interestingly, ablation or blockade of this complex inhibitory system paradoxically leads to hyporesponsive NK cells instead of increased potency (77). Unfortunately, clinical attempts to augment NK cell anti-tumor cytotoxicity through KIR inhibition have been discouraging as there was a marked lack of efficacy and hyporesponsive NK cells (78, 79).

NK cells are also susceptible to TME immunosuppressive soluble and receptor-based inhibition (IL-10, TGF-β, PD-L1) but their ability to be rescued or to blockade these inhibitory signals is far less studied or understood than their T cell counterparts (80). NK cells have shown to be sensitive to hypoxia in the TME as well (81). In addition, large numbers of NK cells have also been required for therapeutic implementation and the collection and culture is not only expensive but time consuming, particularly with traditional cytokine-based expansion methods. The K562 feeder cell lines that have been established have improved the cost and speed of NK cell expansion but it remains a complex process to ensure safety for subsequent human administration. Lastly, NK cells are not clonotypically directed like their adaptive immune cell partners (T- or B cells) and are stereotypically shorter-lived. While longer-lived and memory capable NK cell populations have been identified, they remain a limited subset, requiring additional complexity to subculture, and require additional study before their clinical potential can be fully harnessed (82–84).

We have chosen to develop an autologous T cell-based product, more in line with other groups focused on CD30 CARs. A few clinical trials studying CD30 CAR-T cells have been published to date (16, 17, 85, 86); other studies are ongoing (87). While the results seen with CD30 CAR-Ts has been promising thus far, biAbs offer benefits as an alternative approach. Large quantities of mAbs can be produced, conjugated, and stored long-term until needed. Additionally, biAb therapies are produced without genetic engineering of patient T cells, thus reducing the complexity and costs of therapy production and patient monitoring. CAR-T therapies also come with significant side effects, including the potential for life-long ablation of normal cells expressing the TAA being targeted (such as the B cell aplasia seen in patients treated with CD19 CAR-Ts) (88). Similarly, biAb therapy is a more transient treatment option, as the infused armed T cells lose biAb coating density over time as they replicate in the process of repeated killing (88). Multiple doses of the biAb product can be cryopreserved and administered over time in order to maximize the anti-tumor effect. This approach has been successfully applied in clinical trials of other biAb-armed T cell products, and no dose-limiting toxicities have been observed (49, 62, 63, 89, 90).

In conclusion, CD30 biAb-armed T cell therapy may improve the safety and efficacy of CD30 therapies already in use or currently under development. We have demonstrated that our 8D10 biAb-armed T cells are effective at binding to CD30^+^ tumor cells and mediating cytotoxicity specifically to CD30^high^ cells. These biAb-armed T cells also produce proinflammatory cytokines, indicating strong effector activity and the potential to mediate additional anti-tumor activity localized at the tumor site. Our results suggest that this is a viable immunotherapy strategy for the treatment of CD30^+^ malignancies.

## Data availability statement

The datasets presented in this study can be found in online repositories. The names of the repository/repositories and accession number(s) can be found below: The heavy chain and light chain variable sequences for 8D10 (ABCD_AY477), 10C2 (ABCD_AY478), 12B1 (ABCD_AY479), 13H1 (ABCD_AY480) and 15B8 (ABCD_AY481) are deposited in the ABCD (AntiBodies Chemically Defined Database).

## Ethics statement

The animal study was reviewed and approved by Medical College of Wisconsin Institutional Animal Care and Use Committee.

## Author contributions

MF and RO contributed equally to the study. MF, RO, NS, and JM designed the research and wrote the paper. MF, RO, AT, NS, LL, and JM reviewed and edited the paper. MF, RO, AT, MR, EK, TD, and SG performed experiments. MF, RO, WM, and JM performed data analysis and made the figures. All authors approved the manuscript.

## Supplementary material

Supplementary Figure 1 Binding of novel mAbs to Raji and Raji LV30 cells as assessed by FCM. AC10 and our anti-CD30 mAbs were assessed. Isotype controls to NT Raji (light grey) and Raji LV30 (dark grey) were included for reference. Binding of each antibody to NT Raji cells (black lines) compared to Raji LV30 cells (colored lines) is shown.

Supplementary Figure 2 SPR assays to assess the binding kinetics of CD30 mAbs. **(A)** rhCD30 protein was immobilized on the flow cell of a CM5 sensor chip with a final coupling level of 1000 response units (RU). Shown are sensorgrams for 8D10, 10C2, 12B1, and 15B8 at mAb concentrations of 1.25nM, 2.5nM, 5nM, 10nM and 20nM, and 13H1 and AC10 at mAb concentrations of 0.625nM, 1.25nM, 2.5nM, 5nM and 10nM. No binding was observed to the reference flow cell (data not shown). **(B)** The mAbs 8D10, 10C2, and AC10 were immobilized to 3 separate flow cells of a Protein G sensor chip to an approximate density of 200 RU. RhCD30 protein was injected at concentrations of 2500nM, 1000nM, 500nM, 250nM, and 100nM for the 8D10 and 10C2 flow cells, and 25nM, 10nM, and 5nM for the AC10 flow cell. No binding was observed to a reference isotype control antibody flow cell (data not shown).

Supplementary Figure 3 Distance-based amino acid sequence similarity trees. Similarities between the amino acid sequences of the light chains and heavy chains of our five novel anti-CD30 antibodies and AC10 are illustrated.

Supplementary Figure 4 Representative data for epitope cluster mapping *via* competitive binding. Fluorescently labelled AC10, 8D10, 10C2, 12B1, 13H1, or 15B8 were incubated with CD30^+^ SU-DHL-1 cells that had been blocked with excess unlabeled AC10, 8D10, 10C2, 12B1, 13H1, 15B8, or BerH2 antibody. FCM analysis revealed the extent of binding inhibition associated with each antibody pair. High inhibition (lower MFI) indicates a shared epitope and low inhibition (higher MFI) indicates distinct epitopes.

Supplementary Figure 5 Linear epitope mapping. Comparison of the responses of the mAbs 8D10F10 and AC10 assayed against linear huCD30 peptides at concentrations of 100 µg/ml. Microarray read-outs were obtained with a scanner and image analysis was done with PepSlide^®^ Analyzer.

Supplementary Figure 6 BiAb chemical heteroconjugation. **(A)** Coomassie-stained gel following non-reducing PAGE. OKT3/8D10 and OKT3/10C2 dimers are indicated by red arrows. **(B)** The percentage of monomer, dimer, and multimer present in each preparation of biAb, and the dimer:monomer ratio, determined by densitometry.

Supplementary Figure 7 Cytokine production by biAb-armed T cells. Cytokine production by unarmed and biAb-armed T cells after co-culture with tumor cells for 24 hours. Co-culture conditions included no target (0), a CD30^low^ cell line (Raji), CD30^+^ cell lines (Raji LV30 and RPMI 6666), and a CD30^−^ cell line (OCIAML2). Production of IL-5, IL-10, IL-13, MIP-1α, MIP-1β, and Granzyme B was assessed. Results represent means ± SD of 3 independent experiments (n = 3). * = p < 0.05, ** = p < 0.01, *** = p < 0.001) between the indicated groups, calculated using a two-way ANOVA with Dunnett’s multiple comparisons test.

Supplementary Figure 8 8D10 mAb subclone characterization and conjugation. **(A)** Coomassie-stained gel following non-reducing PAGE of purified 8D10 and 8D10F10 followed by **(B)** conjugation to OKT3. OKT3/8D10 and OKT3/8D10F10 dimers are indicated by red arrowheads.

## References

[1] Gruss HJ DaSilva N Hu ZB Uphoff CC Goodwin RG Drexler HG. Expression and regulation of CD30 ligand and CD30 in human leukemia-lymphoma cell lines. Leukemia (1994) 8:2083–94. 7528856

[2] Durkop H Foss HD Eitelbach F Anagnostopoulos I Latza U Pileri S. Expression of the CD30 antigen in non-lymphoid tissues and cells. J Pathol (2000) 190:613–8. doi: 10.1002/(SICI)1096-9896(200004)190:5<613::AID-PATH559>3.0.CO;2-0 10727988

[3] Kim WY Nam SJ Kim S Kim TM Heo DS Kim CW. Prognostic implications of CD30 expression in extranodal natural killer/T-cell lymphoma according to treatment modalities. Leuk Lymphoma (2015) 56:1778–86. doi: 10.3109/10428194.2014.974048 25288491

[4] Cheng J Zhu H Choi JK. CD30 expression in pediatric neoplasms, study of 585 cases. Pediatr Dev Pathol (2017) 20:191–6. doi: 10.1177/1093526616689185 28521633

[5] van der Weyden CA Pileri SA Feldman AL Whisstock J Prince HM. Understanding CD30 biology and therapeutic targeting: a historical perspective providing insight into future directions. Blood Cancer J (2017) 7:e603. doi: 10.1038/bcj.2017.85 28885612 PMC5709754

[6] Berger GK Gee K Votruba C McBride A Anwer F. Potential application and prevalence of the CD30 (Ki-1) antigen among solid tumors: A focus review of the literature. Crit Rev Oncol Hematol (2017) 113:8–17. doi: 10.1016/j.critrevonc.2017.02.021 28427526

[7] Ellis TM Simms PE Slivnick DJ Jack HM Fisher RI. CD30 is a signal-transducing molecule that defines a subset of human activated CD45RO+ T cells. J Immunol (1993) 151:2380–9. doi: 10.4049/jimmunol.151.5.2380 8103064

[8] National Cancer Institute. Cancer Stat Facts: Hodgkin Lymphoma. Surveillance, Epidemiology, and End Results (SEER) Program (2009-2015). Available at: https://seer.cancer.gov/statfacts/html/hodg.html (Accessed May 2020).

[9] Gerrie AS Power MM Shepherd JD Savage KJ Sehn LH Connors JM. Chemoresistance can be overcome with high-dose chemotherapy and autologous stem-cell transplantation for relapsed and refractory Hodgkin lymphoma. Ann Oncol (2014) 25:2218–23. doi: 10.1093/annonc/mdu387 25149708

[10] Gopal AK Metcalfe TL Gooley TA Pagel JM Petersdorf SH Bensinger WI. High-dose therapy and autologous stem cell transplantation for chemoresistant Hodgkin lymphoma: the Seattle experience. Cancer (2008) 113:1344–50. doi: 10.1002/cncr.23715 PMC2700660 18623377

[11] Villa D Seshadri T Puig N Massey C Tsang R Keating A. Second-line salvage chemotherapy for transplant-eligible patients with Hodgkin’s lymphoma resistant to platinum-containing first-line salvage chemotherapy. Haematologica (2012) 97:751–7. doi: 10.3324/haematol.2011.047670 PMC3342979 22180434

[12] FDA. Brentuximab Vedotin Approval Package. Available at: https://www.accessdata.fda.gov/drugsatfda_docs/nda/2011/125399_adcetris_toc.cfm (Accessed May 2023).

[13] Chen R Gopal AK Smith SE Ansell SM Rosenblatt JD Savage KJ. Five-year survival and durability results of brentuximab vedotin in patients with relapsed or refractory Hodgkin lymphoma. Blood (2016) 128:1562–6. doi: 10.1182/blood-2016-02-699850 PMC5034737 27432875

[14] Castellino SM Pei Q Parsons SK Hodgson D McCarten K Horton T. Brentuximab vedotin with chemotherapy in pediatric high-risk Hodgkin’s lymphoma. N Engl J Med (2022) 387:1649–60. doi: 10.1056/NEJMoa2206660 PMC9945772 36322844

[15] FDA. Adcetris (brentuximab vedotin) . Available at: https://www.accessdata.fda.gov/drugsatfda_docs/label/2018/125388s097lbl.pdf (Accessed May 2020).

[16] Wang CM Wu ZQ Wang Y Guo YL Dai HR Wang XH. Autologous T cells expressing CD30 chimeric antigen receptors for relapsed or refractory Hodgkin lymphoma: an open-label phase I trial. Clin Cancer Res (2017) 23:1156–66. doi: 10.1158/1078-0432.CCR-16-1365 27582488

[17] Ramos CA Ballard B Zhang H Dakhova O Gee AP Mei Z. Clinical and immunological responses after CD30-specific chimeric antigen receptor-redirected lymphocytes. J Clin Invest (2017) 127:3462–71. doi: 10.1172/JCI94306 PMC5669573 28805662

[18] Zhang S Gu C Huang L Wu H Shi J Zhang Z. The third-generation anti-CD30 CAR T-cells specifically homing to the tumor and mediating powerful antitumor activity. Sci Rep (2022) 12:10488. doi: 10.1038/s41598-022-14523-0 35729339 PMC9213494

[19] Wu J Fu J Zhang M Liu D. AFM13: a first-in-class tetravalent bispecific anti-CD30/CD16A antibody for NK cell-mediated immunotherapy. J Hematol Oncol (2015) 8:96. doi: 10.1186/s13045-015-0188-3 26231785 PMC4522136

[20] Hartmann F Renner C Jung W da Costa L Tembrink S Held G. Anti-CD16/CD30 bispecific antibody treatment for Hodgkin’s disease: role of infusion schedule and costimulation with cytokines. Clin Cancer Res (2001) 7:1873–81. 11448899

[21] Lum LG Al-Kadhimi Z. Development and prospects for bispecific antibody-based therapeutics in cancer and other applications. Expert Opin Drug Discov (2008) 3:1081–97. doi: 10.1517/17460441.3.9.1081 23506181

[22] Thakur A Lum LG. “NextGen” Biologics: bispecific antibodies and emerging clinical results. Expert Opin Biol Ther (2016) 16:675–88. doi: 10.1517/14712598.2016.1150996 26848610

[23] Valone FH Kaufman PA Guyre PM Lewis LD Memoli V Deo Y. Phase Ia/Ib trial of bispecific antibody MDX-210 in patients with advanced breast or ovarian cancer that overexpresses the proto-oncogene HER-2/neu. J Clin Oncol (1995) 13:2281–92. doi: 10.1200/JCO.1995.13.9.2281 7545221

[24] Davico Bonino L De Monte LB Spagnoli GC Vola R Mariani M Barone D. Bispecific monoclonal antibody anti-CD3 x anti-tenascin: an immunotherapeutic agent for human glioma. Int J Cancer (1995) 61:509–15. doi: 10.1002/ijc.2910610414 7538978

[25] Bolhuis RL Lamers CH Goey SH Eggermont AM Trimbos JB Stoter G. Adoptive immunotherapy of ovarian carcinoma with bs-MAb-targeted lymphocytes: a multicenter study. Int J Cancer Suppl (1992) 7:78–81. 1428412

[26] Pohl C Denfeld R Renner C Jung W Bohlen H Sahin U. CD30-antigen-specific targeting and activation of T cells *via* murine bispecific monoclonal antibodies against CD3 and CD28: potential use for the treatment of Hodgkin’s lymphoma. Int J Cancer (1993) 54:820–7. doi: 10.1002/ijc.2910540517 7686889

[27] Schneider CA Rasband WS Eliceiri KW. NIH Image to ImageJ: 25 years of image analysis. Nat Methods (2012) 9:671–5. doi: 10.1038/nmeth.2089 PMC5554542 22930834

[28] Scaife M Pacienza N Au BC Wang JC Devine S Scheid E. Engineered human Tmpk fused with truncated cell-surface markers: versatile cell-fate control safety cassettes. Gene Ther (2013) 20:24–34. doi: 10.1038/gt.2011.210 22241175

[29] Sievers F Wilm A Dineen D Gibson TJ Karplus K Li W. Fast, scalable generation of high-quality protein multiple sequence alignments using Clustal Omega. Mol Syst Biol (2011) 7:539. doi: 10.1038/msb.2011.75 21988835 PMC3261699

[30] Sen M Wankowski DM Garlie NK Siebenlist RE Van Epps D LeFever AV. Use of anti-CD3 x anti-HER2/neu bispecific antibody for redirecting cytotoxicity of activated T cells toward HER2/neu+ tumors. J Hematother Stem Cell Res (2001) 10:247–60. doi: 10.1089/15258160151134944 11359672

[31] Wahl AF Klussman K Thompson JD Chen JH Francisco LV Risdon G. The anti-CD30 monoclonal antibody SGN-30 promotes growth arrest and DNA fragmentation in *vitro* and affects antitumor activity in models of Hodgkin’s disease. Cancer Res (2002) 62:3736–42. 12097283

[32] Gruss HJ Boiani N Williams DE Armitage RJ Smith CA Goodwin RG. Pleiotropic effects of the CD30 ligand on CD30-expressing cells and lymphoma cell lines. Blood (1994) 83:2045–56. doi: 10.1182/blood.V83.8.2045.2045 8161776

[33] European Medicines Agency. Assessment report: Adcetris. EMA/702390/2012. Procedure number EMEA/H/C/002455 (2012). Available at: https://www.ema.europa.eu/en/documents/assessment-report/adcetris-epar-public-assessment-report_en.pdf (Accessed May 2020).

[34] Kang L Jiang D Ehlerding EB Barnhart TE Ni D Engle JW. Noninvasive trafficking of brentuximab vedotin and PET imaging of CD30 in lung cancer murine models. Mol Pharm (2018) 15:1627–34. doi: 10.1021/acs.molpharmaceut.7b01168 PMC5920523 29537283

[35] Takeda. A Phase 1/2 Study of brentuximab vedotin (SGN-35) in Pediatric Patients With Relapsed or Refractory Systemic Anaplastic Large-Cell Lymphoma or Hodgkin Lymphoma. Clinical Study Protocol C25002 Amendment 4 (2014). Available at: https://clinicaltrials.gov/ProvidedDocs/88/NCT01492088/Prot_001.pdf (Accessed May 2020).

[36] Francisco JA Risdon G Wahl AF Siegall CB. Recombinant anti-cd30 antibodies and uses thereof. (2002).

[37] Horn-Lohrens O Tiemann M Lange H Kobarg J Hafner M Hansen H. Shedding of the soluble form of CD30 from the Hodgkin-analogous cell line L540 is strongly inhibited by a new CD30-specific antibody (Ki-4). Int J Cancer (1995) 60:539–44. doi: 10.1002/ijc.2910600419 7530238

[38] Ponten F Jirstrom K Uhlen M. The Human Protein Atlas–a tool for pathology. J Pathol (2008) 216:387–93. doi: 10.1002/path.2440 18853439

[39] Choi S Pegues MA Lam N Geldres C Vanasse D Kochenderfer JN. Design and assessment of novel anti-CD30 chimeric antigen receptors with human antigen-recognition domains. Hum Gene Ther (2021) 32:730–43. doi: 10.1089/hum.2020.215 PMC8312022 33287637

[40] Younes A Gopal AK Smith SE Ansell SM Rosenblatt JD Savage KJ. Results of a pivotal phase II study of brentuximab vedotin for patients with relapsed or refractory Hodgkin’s lymphoma. J Clin Oncol (2012) 30:2183–9. doi: 10.1200/JCO.2011.38.0410 PMC3646316 22454421

[41] Pro B Advani R Brice P Bartlett NL Rosenblatt JD Illidge T. Brentuximab vedotin (SGN-35) in patients with relapsed or refractory systemic anaplastic large-cell lymphoma: results of a phase II study. J Clin Oncol (2012) 30:2190–6. doi: 10.1200/JCO.2011.38.0402 22614995

[42] Nathwani N Krishnan AY Huang Q Kim Y Karanes C Smith EP. Persistence of CD30 expression in Hodgkin lymphoma following brentuximab vedotin (SGN-35) treatment failure. Leuk Lymphoma (2012) 53:2051–3. doi: 10.3109/10428194.2012.666543 PMC3808117 22369501

[43] Sotillo E Barrett DM Black KL Bagashev A Oldridge D Wu G. Convergence of acquired mutations and alternative splicing of CD19 enables resistance to CART-19 immunotherapy. Cancer Discovery (2015) 5:1282–95. doi: 10.1158/2159-8290.CD-15-1020 PMC4670800 26516065

[44] Cao Y Lam L. Bispecific antibody conjugates in therapeutics. Adv Drug Delivery Rev (2003) 55:171–97. doi: 10.1016/s0169-409x(02)00178-3 12564976

[45] Thurber GM Schmidt MM Wittrup KD. Antibody tumor penetration: transport opposed by systemic and antigen-mediated clearance. Adv Drug Delivery Rev (2008) 60:1421–34. doi: 10.1016/j.addr.2008.04.012 PMC2820307 18541331

[46] Xenaki KT Oliveira S van Bergen En Henegouwen PMP. Antibody or antibody fragments: implications for molecular imaging and targeted therapy of solid tumors. Front Immunol (2017) 8:1287. doi: 10.3389/fimmu.2017.01287 29075266 PMC5643388

[47] Davol PA Smith JA Kouttab N Elfenbein GJ Lum LG. Anti-CD3 x anti-HER2 bispecific antibody effectively redirects armed T cells to inhibit tumor development and growth in hormone-refractory prostate cancer-bearing severe combined immunodeficient beige mice. Clin Prostate Cancer (2004) 3:112–21. doi: 10.3816/cgc.2004.n.021 15479495

[48] Lum LG Rathore R Cummings F Colvin GA Radie-Keane K Maizel A. Phase I/II study of treatment of stage IV breast cancer with OKT3 x trastuzumab-armed activated T cells. Clin Breast Cancer (2003) 4:212–7. doi: 10.3816/cbc.2003.n.028 14499016

[49] Lum LG Thakur A Choi M Deol A Kondadasula V Schalk D. Clinical and Immune Responses to Anti-CD3 x Anti-EGFR Bispecific Antibody Armed Activated T cells (EGFR BATs) in Pancreatic Cancer Patients. OncoImmunology (2020) 9(1):1773201. doi: 10.1080/2162402X.2020.1773201 32939319 PMC7480816

[50] Chan JK Hamilton CA Cheung MK Karimi M Baker J Gall JM. Enhanced killing of primary ovarian cancer by retargeting autologous cytokine-induced killer cells with bispecific antibodies: a preclinical study. Clin Cancer Res (2006) 12:1859–67. doi: 10.1158/1078-0432.CCR-05-2019 16551871

[51] van de Donk NW Dhimolea E. Brentuximab vedotin. MAbs (2012) 4:458–65. doi: 10.4161/mabs.20230 PMC3499340 22684302

[52] Dubowchik GM Firestone RA Padilla L Willner D Hofstead SJ Mosure K. Cathepsin B-labile dipeptide linkers for lysosomal release of doxorubicin from internalizing immunoconjugates: model studies of enzymatic drug release and antigen-specific in *vitro* anticancer activity. Bioconjug Chem (2002) 13:855–69. doi: 10.1021/bc025536j 12121142

[53] Masters JC Nickens DJ Xuan D Shazer RL Amantea M. Clinical toxicity of antibody drug conjugates: a meta-analysis of payloads. Invest New Drugs (2018) 36:121–35. doi: 10.1007/s10637-017-0520-6 29027591

[54] Vaklavas C Forero-Torres A. Safety and efficacy of brentuximab vedotin in patients with Hodgkin lymphoma or systemic anaplastic large cell lymphoma. Ther Adv Hematol (2012) 3:209–25. doi: 10.1177/2040620712443076 PMC3627331 23606932

[55] Staudacher AH Brown MP. Antibody drug conjugates and bystander killing: is antigen-dependent internalisation required? Br J Cancer (2017) 117:1736–42. doi: 10.1038/bjc.2017.367 PMC5729478 29065110

[56] Brown MP Staudacher AH. Could bystander killing contribute significantly to the antitumor activity of brentuximab vedotin given with standard first-line chemotherapy for Hodgkin lymphoma? Immunotherapy (2014) 6:371–5. doi: 10.2217/imt.14.13 24815777

[57] Masuda S Miyagawa S Sougawa N Sawa Y. CD30-targeting immunoconjugates and bystander effects. Nat Rev Clin Oncol (2015) 12. doi: 10.1038/nrclinonc.2014.159-c1 25734636

[58] Fromm JR McEarchern JA Kennedy D Thomas A Shustov AR Gopal AK. Clinical binding properties, internalization kinetics, and clinicopathologic activity of brentuximab vedotin: an antibody-drug conjugate for CD30-positive lymphoid neoplasms. Clin Lymphoma Myeloma Leuk (2012) 12:280–3. doi: 10.1016/j.clml.2012.01.012 22542449

[59] Duvic M Tetzlaff MT Gangar P Clos AL Sui D Talpur R. Results of a phase II trial of Brentuximab vedotin for CD30+ Cutaneous T-cell lymphoma and lymphomatoid papulosis. J Clin Oncol (2015) 33:3759–65. doi: 10.1200/JCO.2014.60.3787 PMC4737859 26261247

[60] Jacobsen ED Sharman JP Oki Y Advani RH Winter JN Bello CM. Brentuximab vedotin demonstrates objective responses in a phase 2 study of relapsed/refractory DLBCL with variable CD30 expression. Blood (2015) 125:1394–402. doi: 10.1182/blood-2014-09-598763 25573987

[61] Brossart P. The role of antigen spreading in the efficacy of immunotherapies. Clin Cancer Res (2020) 26:4442–7. doi: 10.1158/1078-0432.CCR-20-0305 32357962

[62] Lum LG Thakur A Al-Kadhimi Z Colvin GA Cummings FJ Legare RD. Targeted T-cell therapy in stage IV breast cancer: A phase I clinical trial. Clin Cancer Res (2015) 21:2305–14. doi: 10.1158/1078-0432.CCR-14-2280 PMC4433762 25688159

[63] Lum LG Thakur A Kondadasula SV Al-Kadhimi Z Deol A Tomaszewski EN. Targeting CD138-/CD20+ Clonogenic myeloma precursor cells decreases these cells and induces transferable antimyeloma immunity. Biol Blood Marrow Transplant (2016) 22:869–78. doi: 10.1016/j.bbmt.2015.12.030 PMC6820521 26827660

[64] Thakur A Rathore R Kondadasula SV Uberti JP Ratanatharathorn V Lum LG. Immune T cells can transfer and boost anti-breast cancer immunity. Oncoimmunology (2018) 7:e1500672. doi: 10.1080/2162402X.2018.1500672 30524893 PMC6279339

[65] Vaishampayan U Thakur A Rathore R Kouttab N Lum LG. Phase I study of anti-CD3 x anti-Her2 bispecific antibody in metastatic castrate resistant prostate cancer patients. Prostate Cancer (2015) 2015:285193. doi: 10.1155/2015/285193 25802762 PMC4352947

[66] Dao T Pankov D Scott A Korontsvit T Zakhaleva V Xu Y. Therapeutic bispecific T-cell engager antibody targeting the intracellular oncoprotein WT1. Nat Biotechnol (2015) 33:1079–86. doi: 10.1038/nbt.3349 PMC4600043 26389576

[67] Lum LG Thakur A. Targeting T cells with bispecific antibodies for cancer therapy. BioDrugs (2011) 25:365–79. doi: 10.2165/11595950-000000000-00000 PMC3792709 22050339

[68] Link BK Kostelny SA Cole MS Fusselman WP Tso JY Weiner GJ. Anti-CD3-based bispecific antibody designed for therapy of human B-cell malignancy can induce T-cell activation by antigen-dependent and antigen-independent mechanisms. Int J Cancer (1998) 77:251–6. doi: 10.1002/(sici)1097-0215(19980717)77:2<251::aid-ijc14>3.0.co;2-e 9650561

[69] Zhu M Wu B Brandl C Johnson J Wolf A Chow A. Blinatumomab, a bispecific T-cell engager (BiTE((R))) for CD-19 targeted cancer immunotherapy: clinical pharmacology and its implications. Clin Pharmacokinet (2016) 55:1271–88. doi: 10.1007/s40262-016-0405-4 27209293

[70] Institute of Medicine (US) Committee on Psychosocial Services to Cancer Patients/Families in a Community Setting. Cancer Care for the Whole Patient: Meeting Psychosocial Health Needs (2008). National Academies Press (US. Available at: http://www.ncbi.nlm.nih.gov/books/NBK4015/ (Accessed January 19, 2022).

[71] Mack JW Hilden JM Watterson J Moore C Turner B Grier HE. Parent and physician perspectives on quality of care at the end of life in children with cancer. J Clin Oncol (2005) 23:9155–61. doi: 10.1200/JCO.2005.04.010 16172457

[72] Hartmann F Renner C Jung W Pfreundschuh M. Anti-CD16/CD30 bispecific antibodies as possible treatment for refractory Hodgkin’s disease. Leuk Lymphoma (1998) 31:385–92. doi: 10.3109/10428199809059232 9869203

[73] Reusch U Burkhardt C Fucek I Le Gall F Le Gall M Hoffmann K. A novel tetravalent bispecific TandAb (CD30/CD16A) efficiently recruits NK cells for the lysis of CD30+ tumor cells. MAbs (2014) 6:728–39. doi: 10.4161/mabs.28591 PMC4011917 24670809

[74] Wagner JA Rosario M Romee R Berrien-Elliott MM Schneider SE Leong JW. CD56bright NK cells exhibit potent antitumor responses following IL-15 priming. J Clin Invest (2017) 127:4042–58. doi: 10.1172/JCI90387 PMC5663359 28972539

[75] Romee R Foley B Lenvik T Wang Y Zhang B Ankarlo D. NK cell CD16 surface expression and function is regulated by a disintegrin and metalloprotease-17 (ADAM17). Blood (2013) 121:3599–608. doi: 10.1182/blood-2012-04-425397 PMC3643761 23487023

[76] Long EO. Negative signaling by inhibitory receptors: the NK cell paradigm. Immunol Rev (2008) 224:70–84. doi: 10.1111/j.1600-065X.2008.00660.x 18759921 PMC2587243

[77] Fernandez NC Treiner E Vance RE Jamieson AM Lemieux S Raulet DH. A subset of natural killer cells achieves self-tolerance without expressing inhibitory receptors specific for self-MHC molecules. Blood (2005) 105:4416–23. doi: 10.1182/blood-2004-08-3156 PMC1895026 15728129

[78] Korde N Carlsten M Lee MJ Minter A Tan E Kwok M. A phase II trial of pan-KIR2D blockade with IPH2101 in smoldering multiple myeloma. Haematologica (2014) 99:e81–83. doi: 10.3324/haematol.2013.103085 PMC4040899 24658821

[79] Carlsten M Korde N Kotecha R Reger R Bor S Kazandjian D. Checkpoint inhibition of KIR2D with the monoclonal antibody IPH2101 induces contraction and hyporesponsiveness of NK cells in patients with myeloma. Clin Cancer Res (2016) 22:5211–22. doi: 10.1158/1078-0432.CCR-16-1108 PMC8638787 27307594

[80] Vitale M Cantoni C Pietra G Mingari MC Moretta L. Effect of tumor cells and tumor microenvironment on NK-cell function. Eur J Immunol (2014) 44:1582–92. doi: 10.1002/eji.201344272 24777896

[81] Parodi M Raggi F Cangelosi D Manzini C Balsamo M Blengio F. Hypoxia modifies the transcriptome of human NK cells, modulates their immunoregulatory profile, and influences NK cell subset migration. Front Immunol (2018) 9:2358. doi: 10.3389/fimmu.2018.02358 30459756 PMC6232835

[82] Sun JC Beilke JN Lanier LL. Adaptive immune features of natural killer cells. Nature (2009) 457:557–61. doi: 10.1038/nature07665 PMC2674434 19136945

[83] Vivier E Ugolini S. Regulatory natural killer cells: new players in the IL-10 anti-inflammatory response. Cell Host Microbe (2009) 6:493–5. doi: 10.1016/j.chom.2009.12.001 20006835

[84] Guma M Angulo A Vilches C Gomez-Lozano N Malats N Lopez-Botet M. Imprint of human cytomegalovirus infection on the NK cell receptor repertoire. Blood (2004) 104:3664–71. doi: 10.1182/blood-2004-05-2058 15304389

[85] Zhang P Yang X Cao Y Wang J Zhou M Chen L. Autologous stem cell transplantation in tandem with Anti-CD30 CAR T-cell infusion in relapsed/refractory CD30(+) lymphoma. Exp Hematol Oncol (2022) 11:72. doi: 10.1186/s40164-022-00323-9 36253833 PMC9578248

[86] Ramos CA Grover NS Beaven AW Lulla PD Wu MF Ivanova A. Anti-CD30 CAR-T cell therapy in relapsed and refractory Hodgkin lymphoma. J Clin Oncol (2020) 38:3794–804. doi: 10.1200/JCO.20.01342 PMC7655020 32701411

[87] Grover NS Savoldo B. Challenges of driving CD30-directed CAR-T cells to the clinic. BMC Cancer (2019) 19:203. doi: 10.1186/s12885-019-5415-9 30841880 PMC6404322

[88] Grabert RC Cousens LP Smith JA Olson S Gall J Young WB. Human T cells armed with Her2/neu bispecific antibodies divide, are cytotoxic, and secrete cytokines with repeated stimulation. Clin Cancer Res (2006) 12:569–76. doi: 10.1158/1078-0432.CCR-05-2005 16428502

[89] Lum LG Thakur A Liu Q Deol A Al-Kadhimi Z Ayash L. CD20-targeted T cells after stem cell transplantation for high risk and refractory non-Hodgkin’s lymphoma. Biol Blood Marrow Transplant (2013) 19:925–33. doi: 10.1016/j.bbmt.2013.03.010 PMC3794673 23529012

[90] Lum LG Thakur A Pray C Kouttab N Abedi M Deol A. Multiple infusions of CD20-targeted T cells and low-dose IL-2 after SCT for high-risk non-Hodgkin’s lymphoma: a pilot study. Bone Marrow Transplant (2014) 49:73–9. doi: 10.1038/bmt.2013.133 PMC3891305 24056738

