# Supplementary figures and images for: Novel anti-CD30/CD3 bispecific antibodies activate human T cells and mediate potent anti-tumor activity
Source: Front Immunol. 2023 Aug 14;14:1225610. doi: 10.3389/fimmu.2023.1225610 (PMC10461807; doi:10.3389/fimmu.2023.1225610)

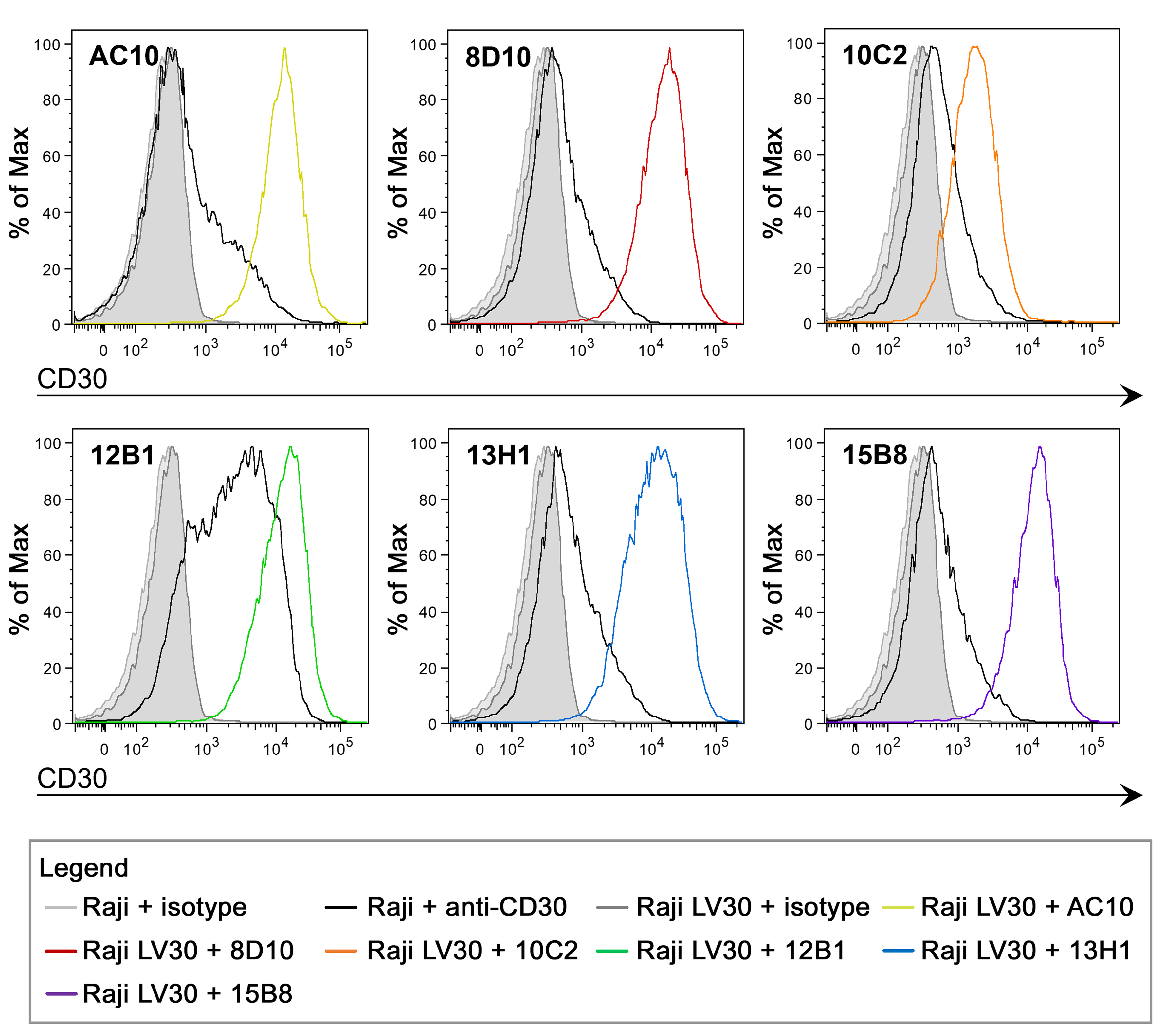

Supplement: Supplementary Figure 1 — Binding of novel mAbs to Raji and Raji LV30 cells as assessed by FCM. AC10 and our anti-CD30 mAbs were assessed. Isotype controls to NT Raji (light grey) and Raji LV30 (dark grey) were included for reference. Binding of each antibody to NT Raji cells (black lines) compared to Raji LV30 cells (colored lines) is shown. [file Image_1.tif]

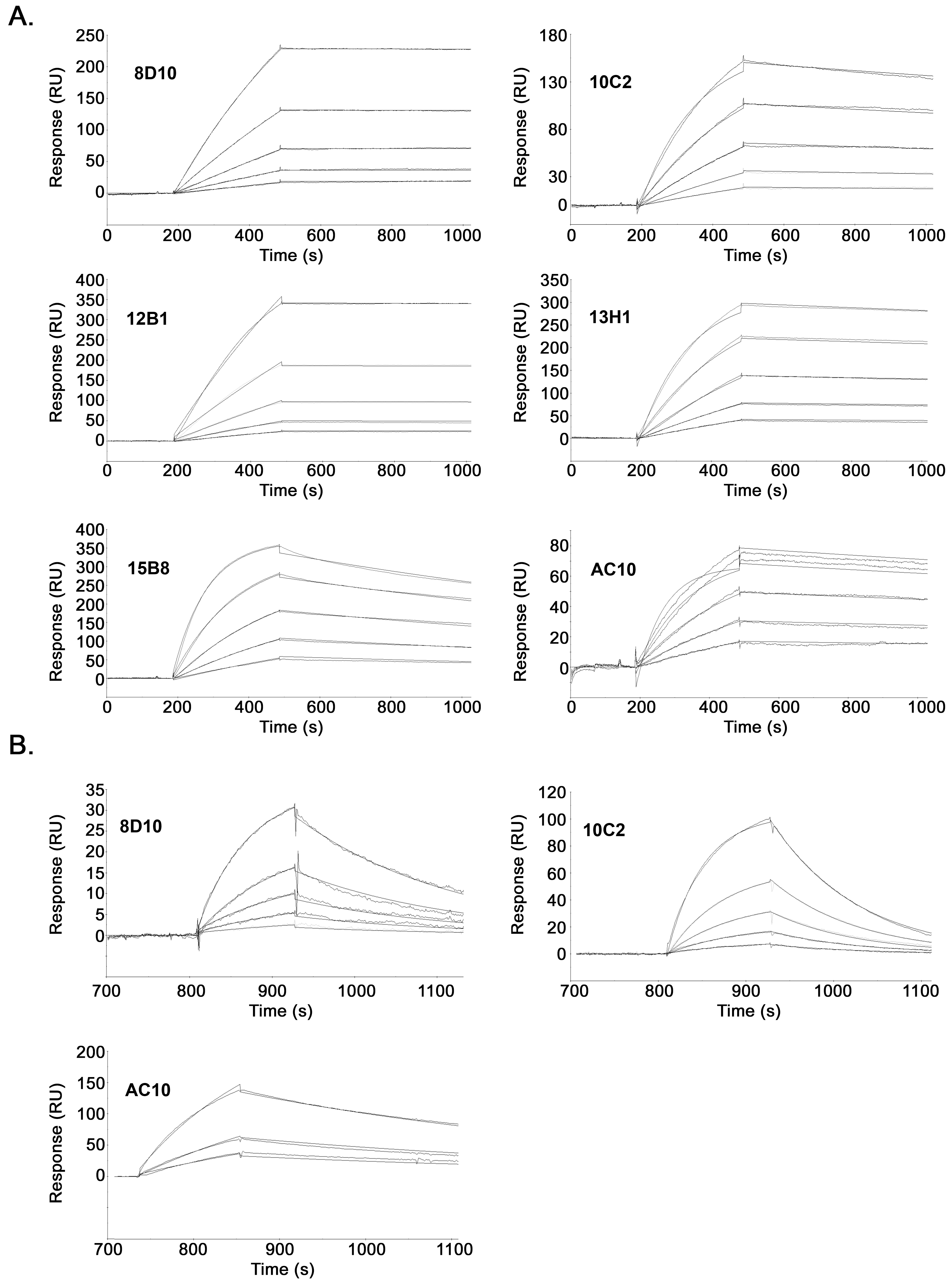

Supplement: Supplementary Figure 2 — SPR assays to assess the binding kinetics of CD30 mAbs. (A) rhCD30 protein was immobilized on the flow cell of a CM5 sensor chip with a final coupling level of 1000 response units (RU). Shown are sensorgrams for 8D10, 10C2, 12B1, and 15B8 at mAb concentrations of 1.25nM, 2.5nM, 5nM, 10nM and 20nM, and 13H1 and AC10 at mAb concentrations of 0.625nM, 1.25nM, 2.5nM, 5nM and 10nM. No binding was observed to the reference flow cell (data not shown). (B) The mAbs 8D10, 10C2, and AC10 were immobilized to 3 separate flow cells of a Protein G sensor chip to an approximate density of 200 RU. RhCD30 protein was injected at concentrations of 2500nM, 1000nM, 500nM, 250nM, and 100nM for the 8D10 and 10C2 flow cells, and 25nM, 10nM, and 5nM for the AC10 flow cell. No binding was observed to a reference isotype control antibody flow cell (data not shown). [file Image_2.tif]

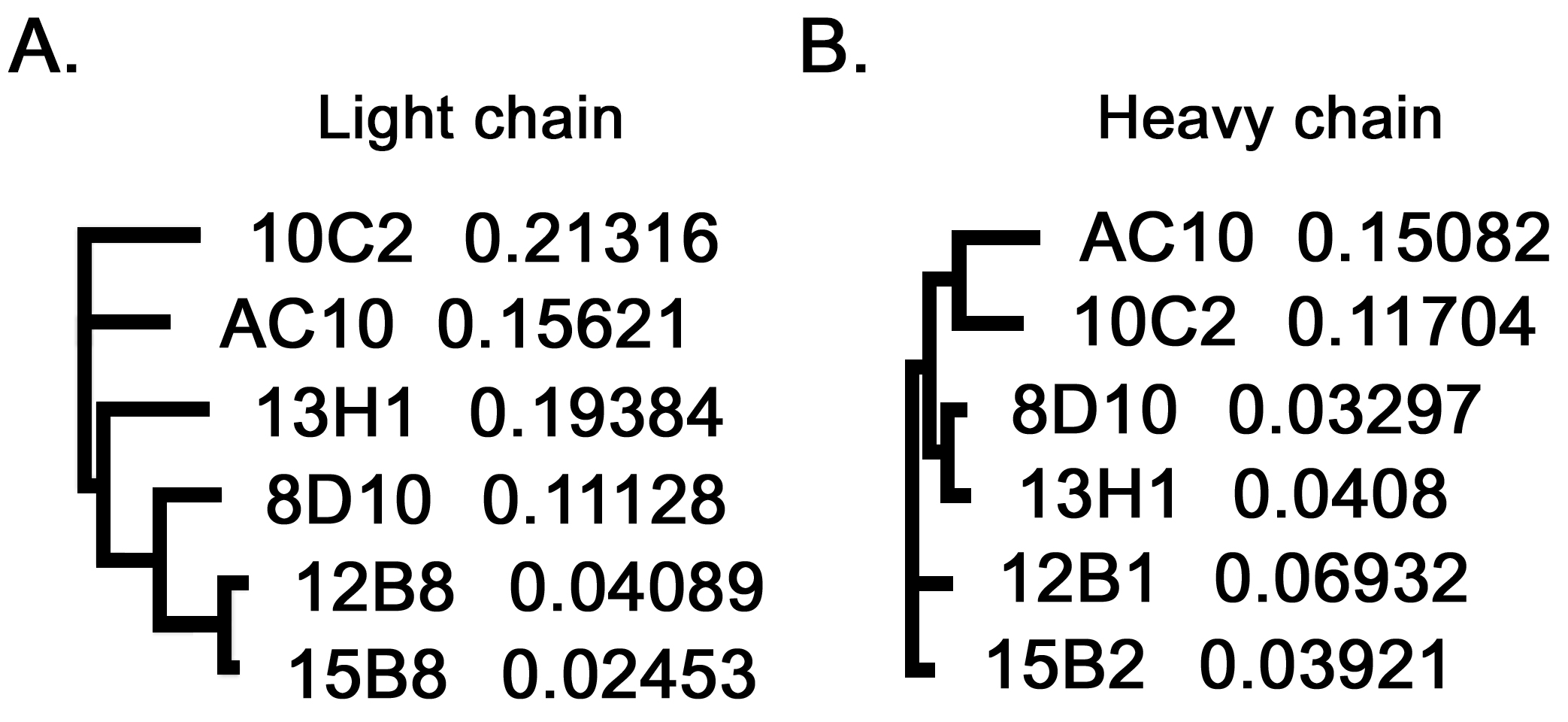

Supplement: Supplementary Figure 3 — Distance-based amino acid sequence similarity trees. Similarities between the amino acid sequences of the light chains and heavy chains of our five novel anti-CD30 antibodies and AC10 are illustrated. [file Image_3.tif]

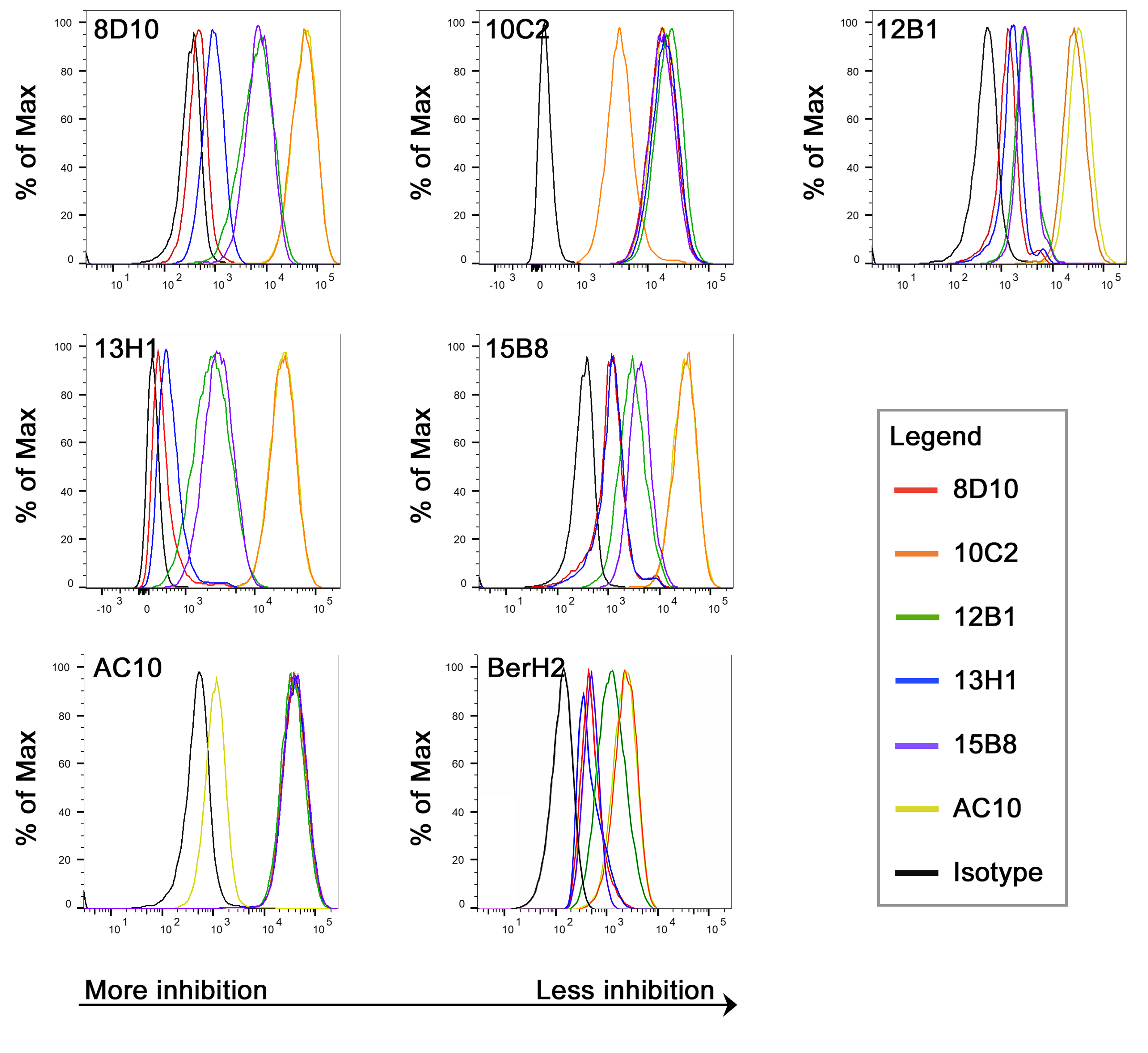

Supplement: Supplementary Figure 4 — Representative data for epitope cluster mapping via competitive binding. Fluorescently labelled AC10, 8D10, 10C2, 12B1, 13H1, or 15B8 were incubated with CD30+ SU-DHL-1 cells that had been blocked with excess unlabeled AC10, 8D10, 10C2, 12B1, 13H1, 15B8, or BerH2 antibody. FCM analysis revealed the extent of binding inhibition associated with each antibody pair. High inhibition (lower MFI) indicates a shared epitope and low inhibition (higher MFI) indicates distinct epitopes. [file Image_4.tif]

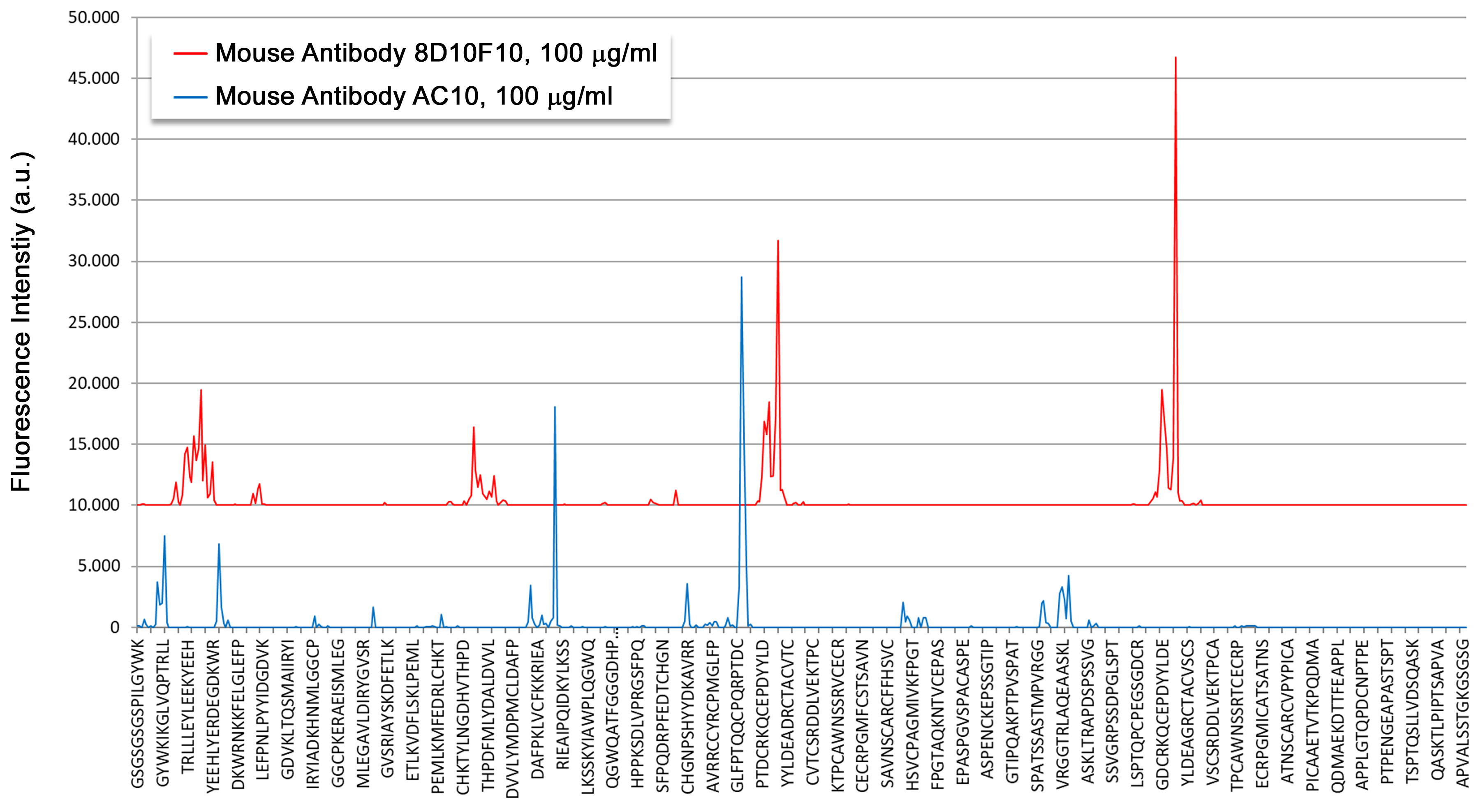

Supplement: Supplementary Figure 5 — Linear epitope mapping. Comparison of the responses of the mAbs 8D10F10 and AC10 assayed against linear huCD30 peptides at concentrations of 100 µg/ml. Microarray read-outs were obtained with a scanner and image analysis was done with PepSlide® Analyzer. [file Image_5.tif]

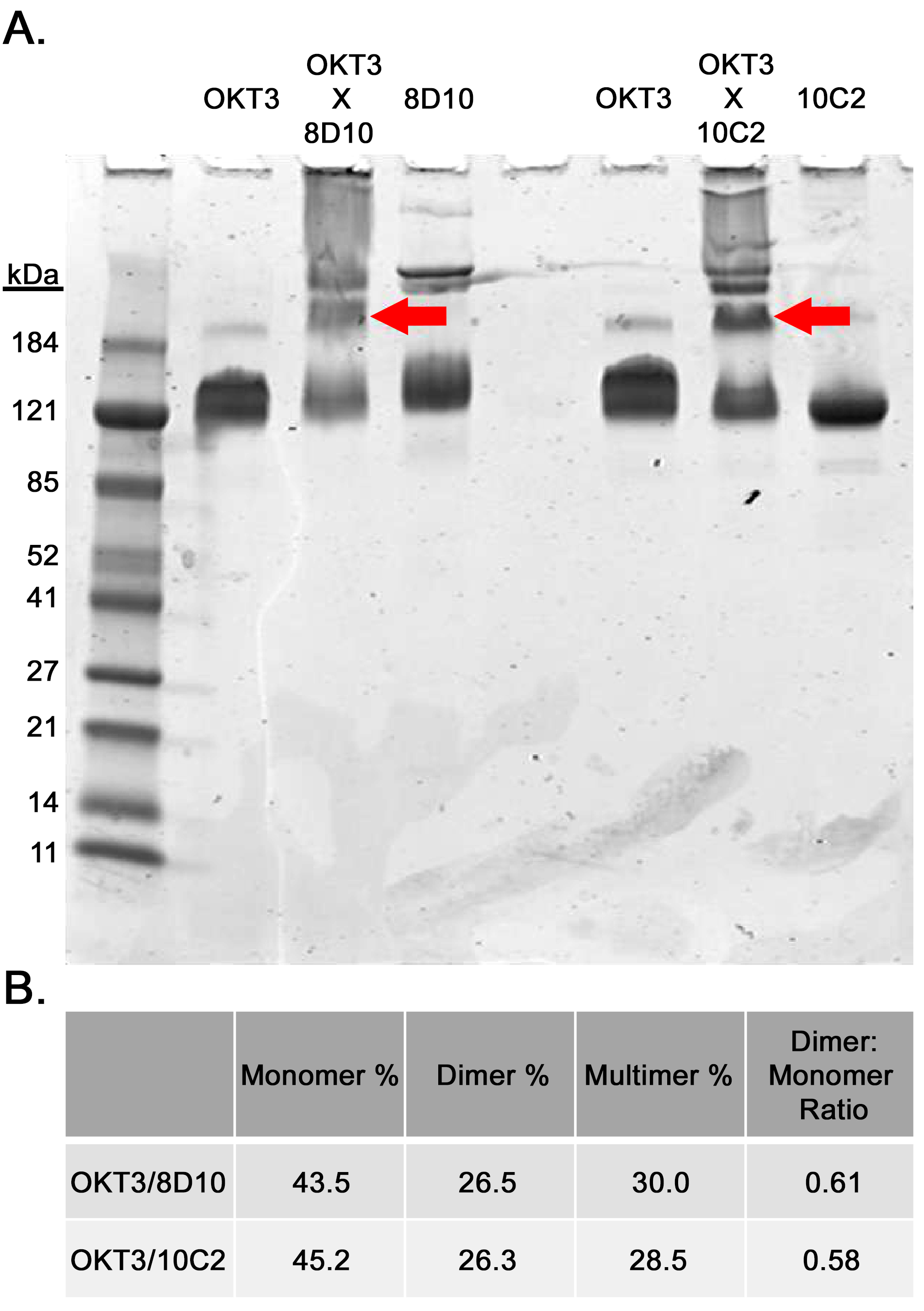

Supplement: Supplementary Figure 6 — BiAb chemical heteroconjugation. (A) Coomassie-stained gel following non-reducing PAGE. OKT3/8D10 and OKT3/10C2 dimers are indicated by red arrows. (B) The percentage of monomer, dimer, and multimer present in each preparation of biAb, and the dimer:monomer ratio, determined by densitometry. [file Image_6.jpeg]

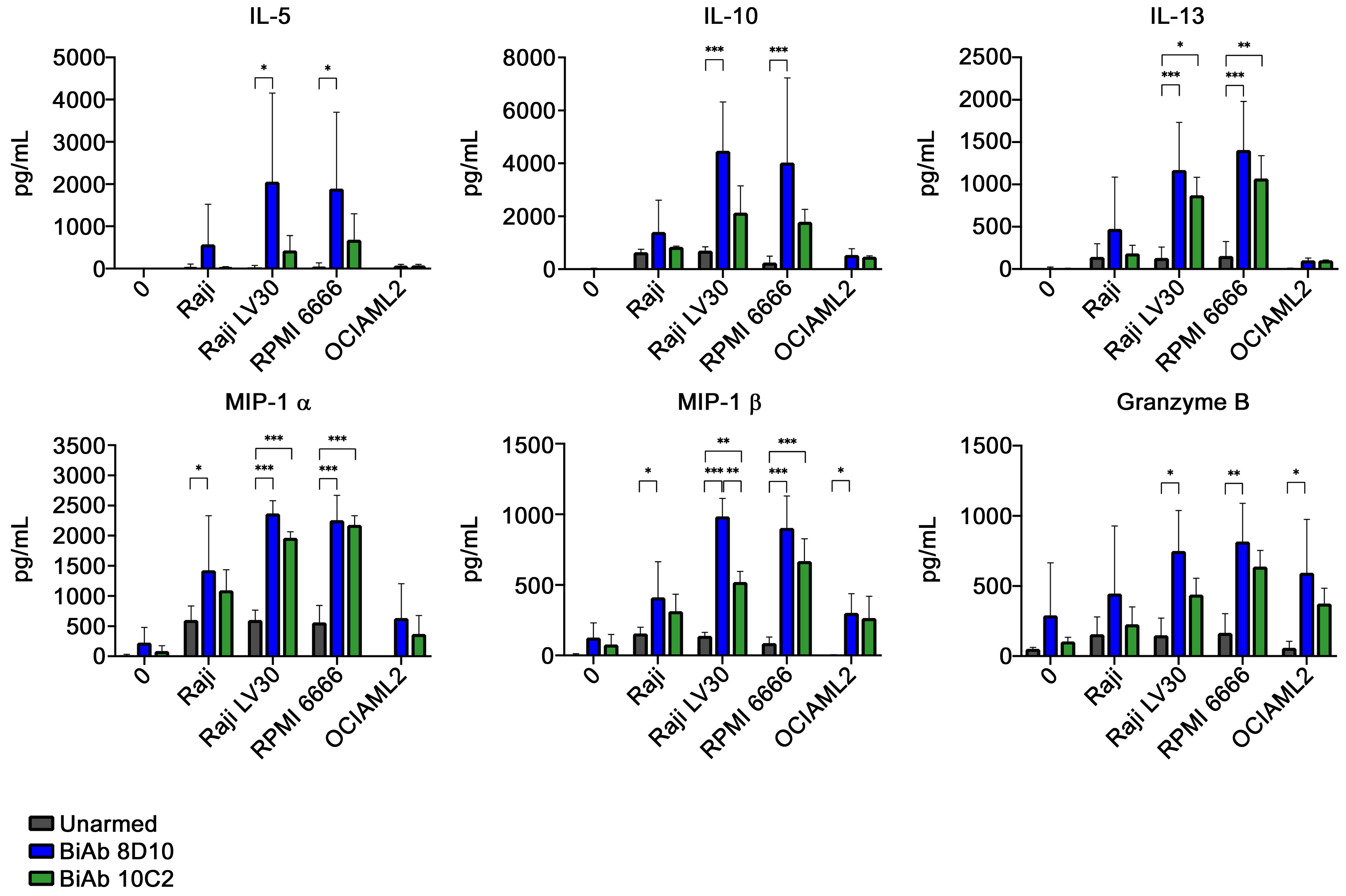

Supplement: Supplementary Figure 7 — Cytokine production by biAb-armed T cells. Cytokine production by unarmed and biAb-armed T cells after co-culture with tumor cells for 24 hours. Co-culture conditions included no target (0), a CD30low cell line (Raji), CD30+ cell lines (Raji LV30 and RPMI 6666), and a CD30− cell line (OCIAML2). Production of IL-5, IL-10, IL-13, MIP-1α, MIP-1β, and Granzyme B was assessed. Results represent means ± SD of 3 independent experiments (n = 3). * = p < 0.05, ** = p < 0.01, *** = p < 0.001) between the indicated groups, calculated using a two-way ANOVA with Dunnett’s multiple comparisons test. [file Image_7.jpg]

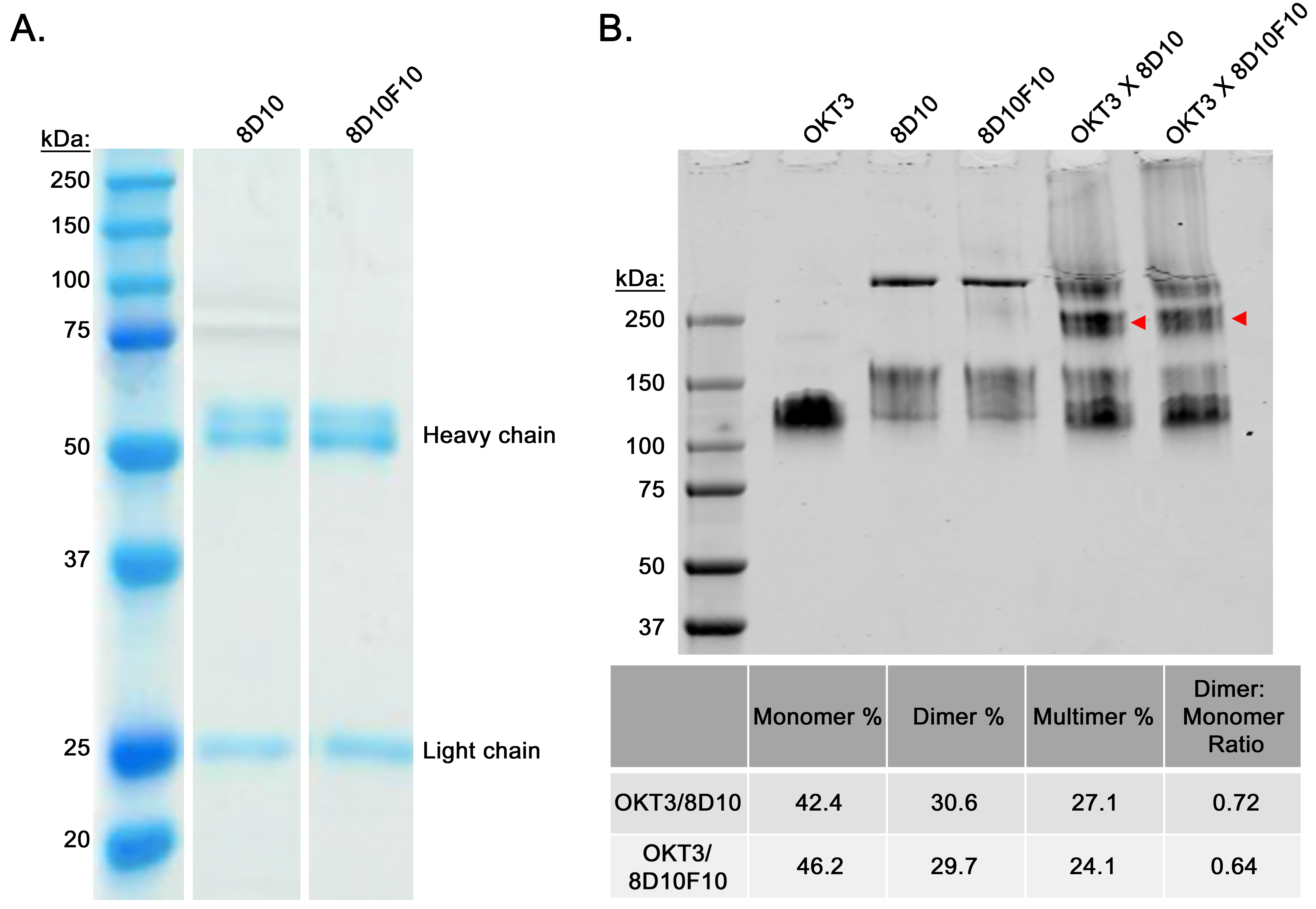

Supplement: Supplementary Figure 8 — 8D10 mAb subclone characterization and conjugation. (A) Coomassie-stained gel following non-reducing PAGE of purified 8D10 and 8D10F10 followed by (B) conjugation to OKT3. OKT3/8D10 and OKT3/8D10F10 dimers are indicated by red arrowheads. [file Image_8.jpg]
