# Supplementary material for: Novel anti-CD30/CD3 bispecific antibodies activate human T cells and mediate potent anti-tumor activity
Source: Front Immunol. 2023 Aug 14;14:1225610. doi: 10.3389/fimmu.2023.1225610 (PMC10461807; doi:10.3389/fimmu.2023.1225610)
Supplement: Supplementary file 9 [file Table_1.docx]

**Supplemental Table 1:** Affinity constants for the interaction of immobilized anti-CD30 mAbs with huCD30 protein.

| **Antibody** | **K_A_ (M^-1^)** | **k_a_ (Ms^-1^)** | **K_D_** | **k_d_ (s^-1^)** |
| --- | --- | --- | --- | --- |
| **8D10** | 8.78x10^5^ | 4.53x10^3^ | 1.14 μM | 5.23x10^-3^ |
| **10C2** | 5.79x10^5^ | 5.88x10^3^ | 1.76 μM | 10.1x10^-3^ |
| **AC10** | 9.03x10^7^ | 1.82x10^5^ | 11.1 nM | 2.02x10^-3^ |
