# Supplementary material for: Novel anti-CD30/CD3 bispecific antibodies activate human T cells and mediate potent anti-tumor activity
Source: Front Immunol. 2023 Aug 14;14:1225610. doi: 10.3389/fimmu.2023.1225610 (PMC10461807; doi:10.3389/fimmu.2023.1225610)
Supplement: Supplementary file 10 [file Table_2.docx]

**Supplemental Table 2**: V_L_ and V_H_ amino acid sequences for all five novel anti-CD30 mAbs

|  | VL Sequence | VH Sequence |
| --- | --- | --- |
| **8D10** | DIVMTQSPASQSASLGESVTITCLASQTIGTWLAWYQQKPGKSPQFLIYAATSLADGVPSRFSGSGSGTKFSFKISSLQAEDFVSYYCQQLYSTPFTFGGGTKLEIK | QVQLQESGTELVKPGASVKLSCKASGYTFTSYWMHWMKQRPGQGLEWIGNINPSNGGTNYNEKFKNKATLTVDKSSSTAYMQLSSLTSEDSAVYYCARRDYYYGSSYGFDVWGTGTTVTVSS |
| **10C2** | DIVLTQTPLTLSVTIGQPASISCKSNQSLLDSYGKTYLNWLLQRPGQSPKRLIYLVSKLDSGVPDRFTGSGSGTDFTLKISRVEAEDLGVYYCWQGTHFPRTFGGGTKLEIK | QVQLEQSGPVLVKPGASVKMSCKASGYTFTDYYMNWVKQSHGKSLEWIGVINPYNGGTSYNQKFKGKATLTVDKSSSTACMELNCLTSEDSAVYYCTLGAYWGQGTSVTVSS |
| **12B1** | DIVMTQTTASLSTSVGETVTITCRASGNLHSYLTWYQQKQGKSPQLLVYNAKTLADGVPSRFSGSGSGTQYSLKIDSLQPEDFGSYYCQHFWTTPFTFGSGTKLEIK | EVKLEESGTELVKPGASVKLSCKASGYTFTSYWMHWVKQRPGQGLEWIGNINPTNGGTNYNEKFKSKATLTVDKSSRTAYMQLSSLTSGDSAVYYCARRDFITTSGFAYWGQGTLVTVSA |
| **13H1** | DIVMTQTPKSMSMSVGERVTLSCKASENVGTYVSWYQQKPEQSPKVLIYGASNRFTGVPDRFTGSGSATDFTLTISSVQTEDLADYHCGQSYSYPLTFGAGTKLELK | QVQLQQSGTELVKPGASVKLSCKASGHTFTSYWMHWVKQRPGQGLEWIGNINPSNGGTNYNEKFKSKATLTVDKSSSTAYMQLSSLTSEDSAVYYCARRGYYGSSSYWSFDVWGTGTTVTVSS |
| **15B8** | DIVMTQTPASLSASVGETVTITCRASGNIHNYLAWYQQKQGKSPQLLVYNAKTLADGVPSRFSGSGSGTQYSLKINSLQPEDFGSYYCQHFWSTPFTFGSGTKLEIK | QVQLEQSGTELVKPGASVKLSCKASGYTFTSYWMHWVKQRPGQGLEWIGNINPSNGGTNYNEKFKSKATLTVDKSSSTAYMQLSSLTSEDSAIYYCARRNNYYASSPFAYWGQGTLVSVSA |
