# Supplementary material for: Novel anti-CD30/CD3 bispecific antibodies activate human T cells and mediate potent anti-tumor activity
Source: Front Immunol. 2023 Aug 14;14:1225610. doi: 10.3389/fimmu.2023.1225610 (PMC10461807; doi:10.3389/fimmu.2023.1225610)
Supplement: Supplementary file 11 [file Table_3.docx]

**Supplemental Table 3**: HH tumor size (mm^3^). Day of ulceration in **bold**

|  | Mouse | Day 3 | 7 | 10 | 14 | 17 | 21 | 24 | 28 |
| --- | --- | --- | --- | --- | --- | --- | --- | --- | --- |
| **PBS** | 1 | 0 | 38.8 | 87.8 | 267.7 | 470.5 | 647.1 | **1291.3** |  |
|  | 2 | 0 | 0 | 99.1 | 228.2 | 347.6 | 583.2 | 1093.8 | **1926.7** |
|  | 3 | 0 | 0 | 127.0 | 178.8 | 181.3 | 327.7 | 505.1 | 1091.7 |
|  | 4 | 56.0 | 55.9 | 53.7 | 143.7 | 336.0 | 665.5 | **1238.9** |  |
|  | 5 | 52.2 | 75.8 | 124.9 | 363.4 | 705.6 | 1238.2 | **1790.3** |  |
|  | 6 | 111.6 | 61.2 | 139.4 | 314.9 | 624.2 | 1069.9 | 1642.5 | **1910.0** |
|  | 7 | 29.7 | 41.5 | 107.6 | 222.3 | 405.0 | 557.0 | 1166.6 | **1417.2** |
|  | 8 | 0 | 0 | 0 | 0 | 243.1 | 389.2 | 721.3 | 1134.8 |
| **UnArmed** | 1 | 0 | 48.4 | 129.0 | 278.8 | 357.4 | 585.0 | 1017.7 | **1608.8** |
|  | 2 | 0 | 58.8 | 142.8 | 256.6 | 320.3 | 616.5 | 1238.2 | **1666.6** |
|  | 3 | 0 | 44.6 | 118.9 | 114.9 | 156.8 | 375.5 | 335.2 | 627.3 |
|  | 4 | 0 | 77.1 | 109.7 | 209.2 | 355.7 | 617.4 | **930.3** |  |
|  | 5 | 55.2 | 71.7 | 99.1 | 166.6 | 238.5 | 629.7 | **1150.0** |  |
|  | 6 | 77.4 | 107.6 | 86.0 | 328.7 | 611.1 | 804.4 | **1447.3** |  |
|  | 7 | 87.5 | 59.6 | 124.1 | 289.3 | 598.2 | 794.7 | 1078.1 | **1670.0** |
|  | 8 | 48.6 | 81.7 | 137.7 | 245.0 | 450.0 | 741.6 | 1119.3 | **1734.6** |
| **8D10 biAb** | 1 | 0 | 44.4 | 117.1 | 225.0 | 367.1 | 670.6 | 1191.5 | 1346.7 |
|  | 2 | 0 | 0 | 94.0 | 67.1 | 175.1 | 314.8 | 367.7 | 398.8 |
|  | 3 | 0 | 28.2 | 49.7 | 45.6 | 117.1 | 233.9 | 435.7 | 545.7 |
|  | 4 | 43.6 | 62.4 | 139.4 | 299.5 | 419.7 | 692.7 | 1247.4 | **1399.7** |
|  | 5 | 28.7 | 100.8 | 128.4 | 328.1 | 474.0 | 583.4 | 953.8 | **1523.5** |
|  | 6 | 47.2 | 49.7 | 143.7 | 142.9 | 227.3 | 258.6 | 393.5 | 951.7 |
|  | 7 | 54.1 | 35.1 | 94.0 | 157.1 | 356.4 | 605.7 | 699.8 | 1435.8 |
|  | 8 | 71.1 | 102.3 | 131.0 | 188.2 | 278.2 | 583.2 | 804.4 | 1398.6 |
